# D-A-D Compounds Combining Dithienopyrrole Donors and Acceptors of Increasing Electron-Withdrawing Capability: Synthesis, Spectroscopy, Electropolymerization, and Electrochromism

**DOI:** 10.1021/acs.jpcb.2c01772

**Published:** 2022-05-26

**Authors:** Renata Rybakiewicz-Sekita, Petr Toman, Roman Ganczarczyk, Jakub Drapala, Przemyslaw Ledwon, Marzena Banasiewicz, Lukasz Skorka, Anna Matyjasiak, Malgorzata Zagorska, Adam Pron

**Affiliations:** † Faculty of Chemistry, Warsaw University of Technology, Noakowskiego 3, 00-664 Warsaw, Poland; ‡ Faculty of Mathematics and Natural Sciences. School of Sciences, Institute of Chemical Sciences, Cardinal Stefan Wyszynski University in Warsaw, Woycickiego 1/3, 01-815 Warsaw, Poland; § Institute of Macromolecular Chemistry, Academy of Sciences of the Czech Republic, Heyrovsky Sq. 2, 162 06 Prague 6, Czech Republic; ∥ Faculty of Chemistry, Silesian University of Technology, Strzody 9, 44-100 Gliwice, Poland; ⊥ Institute of Physics, Polish Academy of Sciences, Al. Lotnikow 32/44, 02-668 Warsaw, Poland

## Abstract

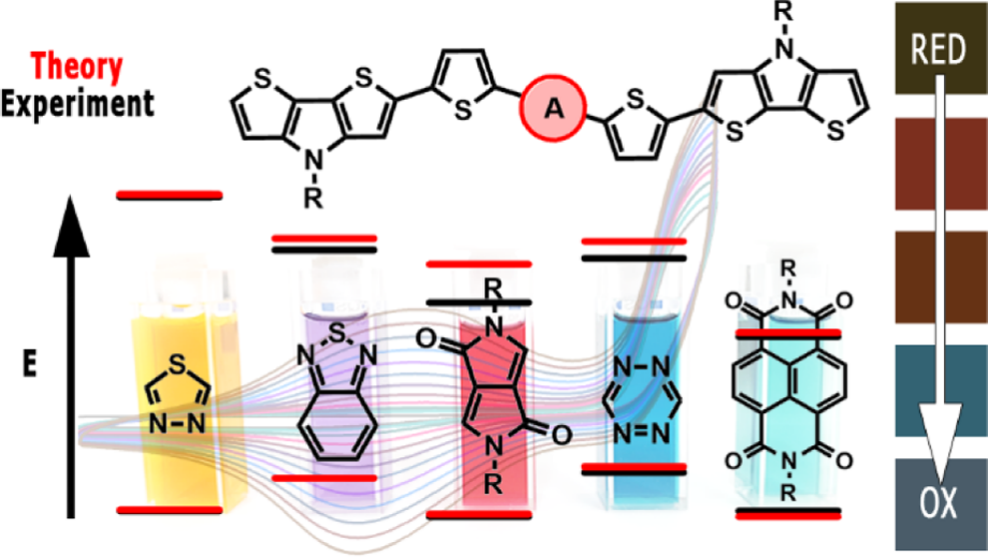

Five **D-π-A-π-D** compounds consisting of the same donor unit (dithieno[3,2-*b*:2′,3′-*d*]pyrrole, **DTP**), the same π-linker (2,5-thienylene), and different acceptors of increasing electron-withdrawing ability (1,3,4-thiadiazole (**TD**), benzo[*c*][1,2,5]thiadiazole (**BTD**), 2,5-dihydropyrrolo[3,4-*c*]pyrrole-1,4-dione (**DPP**), 1,2,4,5-tetrazine (**TZ**), and benzo[*lmn*][3,8]phenanthroline-1,3,6,8(2*H*,7*H*)-tetraone (**NDI**)) were synthesized. **DTP-TD**, **DTP-BTD**, and **DTP-DPP** turned out to be interesting luminophores emitting either yellow (**DTP-TD)** or near-infrared (**DTP-BTD** and **DTP-DPP**) radiation in dichloromethane solutions. The emission bands were increasingly bathochromically shifted with increasing solvent polarity. Electrochemically determined electron affinities (|EA|s) were found to be strongly dependent on the nature of the acceptor changing from 2.86 to 3.84 eV for **DTP-TD** and **DTP-NDI**, respectively, while the ionization potential (IP) values varied only weakly. Experimental findings were strongly supported by theoretical calculations, which correctly predicted the observed solvent dependence of the emission spectra. Similarly, the calculated IP and EA values were in excellent agreement with the experiment. **DTP-TD**, **DTP-BTD**, **DTP-TZ**, and **DTP-NDI** could be electropolymerized to yield polymers of very narrow electrochemical band gap and characterized by redox states differing in color coordinates and lightness. **Poly(DTP-NDI)** and **poly(DTP-TD)** showed promising electrochromic behavior, not only providing a rich color palette in the visible but also exhibiting near-infrared (NIR) electrochromism.

## 1 Introduction

In the past two decades, the synthesis of low and high molecular mass electroactive compounds has attracted significant research interest, which has led to the elaboration of several types of luminescent and/or semiconducting materials suitable for applications in organic electronics and related fields.^1,2^ Among them, donor–acceptor (D-A) compounds deserve special attention. One of their specific features is the possibility of controllable tuning of their redox properties (expressed by the ionization potential (IP) and electron affinity (EA)) as well as their optical properties (*e.g.*, photo- and electroluminescence) by varying D-A interactions between electron-rich and electron-deficient parts of the designed molecules (macromolecules). This approach led to the elaboration of a plethora of D-A electroactive compounds.^3−6^ In particular, molecules (macromolecules) containing dithieno[3,2-*b*:2′,3′-*d*]pyrrole (**DTP**) or its derivatives^7,8^ distinguish themselves by exhibiting promising properties in view of their applications in electronics, optoelectronics, and in electrochromic devices. Dithieno[3,2-b:2′,3′-*d*]pyrrole-based D-A materials were tested as components of active layers in various types of organic field-effect transistors (OFETs).^9−13^ Their electrical transport properties were also exploited in perovskite-type photovoltaic cells, where they were used as charge carrier transporting materials.^14−16^ Band gap tuning resulted in the preparation of several narrow-band gap high^17,18^ and low^19^ molecular mass compounds serving as donor components in bulk heterojunction (BHJ) organic photovoltaic cells (OPVs) containing fullerene acceptors. In a different approach, **DTP** units were used as building blocks of non-fullerene acceptors in the same type of cells.^20−22^ They were also applied as dyes in dye-sensitized photovoltaic cells.^23−25^ Several organic light-emitting diodes (OLEDs) were reported, in which compounds containing dithieno[3,2-*b*:2′,3′-*d*]pyrrole units served as electroluminophores.^26−28^ Electrochromic properties of small molecules^29^ and high molecular mass compounds containing dithienopyrrole moieties^30−32^ were also exploited.

**Scheme 1:**
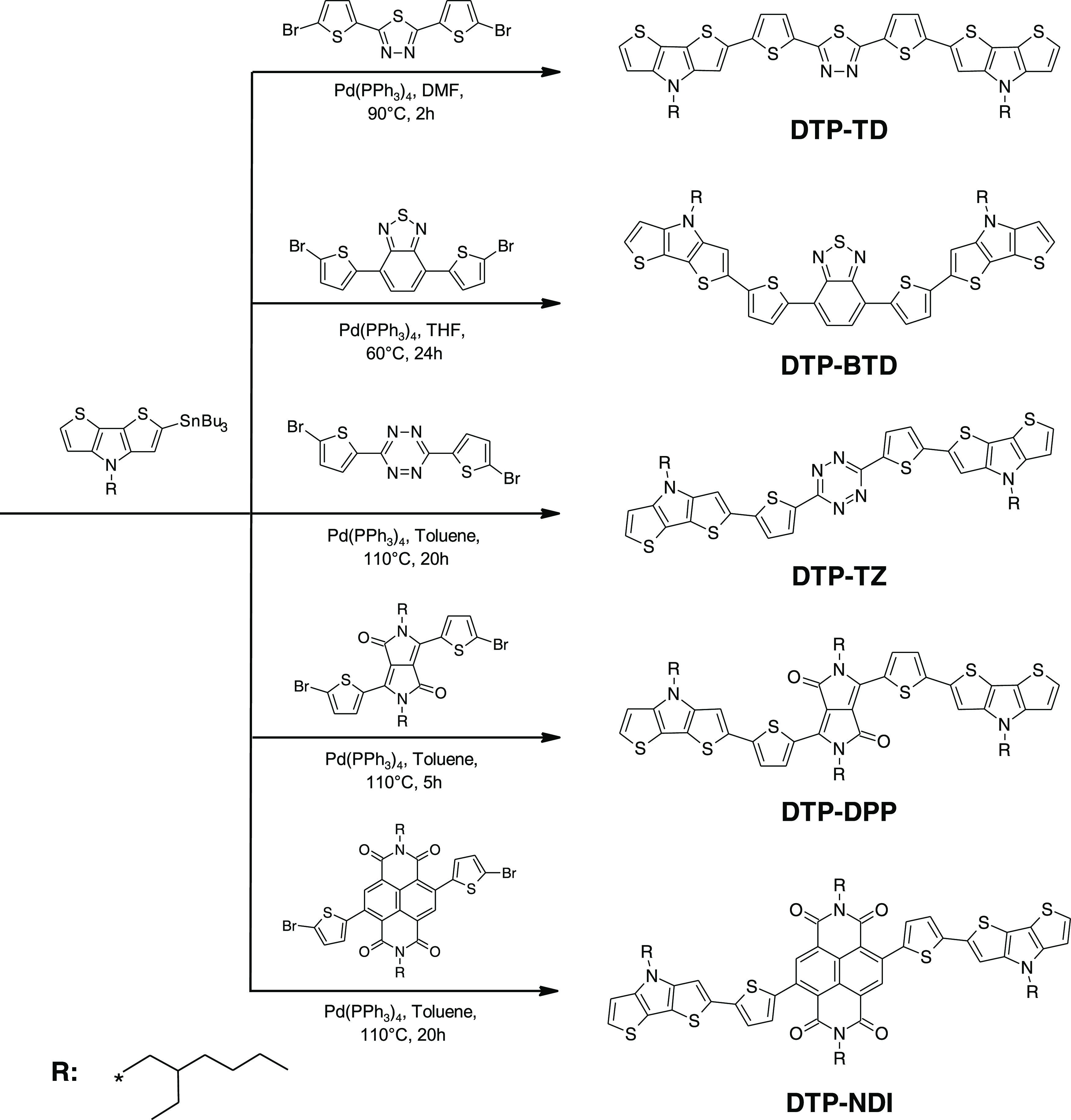
Synthetic Route Leading to the Studied Compounds

In this paper, we present a systematic study on five new D-π-A-π-D compounds consisting of dithieno[3,2-*b*:2′,3′-*d*]pyrrole donors connected *via* 2,5-thienylene linkers to the central acceptor unit of varying electron-withdrawing strengths (Scheme 1). We clearly demonstrate the effect of this central unit on the redox and spectroscopic properties of the synthesized compounds. The obtained experimental results are supported by detailed quantum chemical calculations. Additionally, electrochemical polymerization of the new compounds is investigated, which in four cases yields low-band-gap macromolecular compounds. D-A polymers, including those containing dithieno[3,2-*b*:2′,3′-*d*]pyrrole donors,^17^ frequently exhibit several reversible redox states strongly differing in their spectra both in the UV–visible and near-infrared (NIR) regions,^33^ and for this reason, they are very well suited for electrochromic applications. There are two principal domains in which electrochromic materials are applied. Polymers that undergo electrochemically induced spectral changes in the visible region can be used in different types of passive displays such as electrochromic windows.^34^ Those that exhibit electrochromic activity in the near-infrared range of the spectrum find applications as components of smart windows modulating the heat flow related to NIR radiation.^35^ This modulation is important in telecommunication technologies that work with NIR wavelengths, such as optical fiber communication systems and detectors. Finally, a new direction in this domain of research has been emerging, namely, electrofluorochromism of conjugated molecules, for example, tetrazine derivatives, in which emitted light can be modulated through electrical excitations.^36,37^ In this paper, we demonstrate that the new low-band-gap polymers are especially suitable candidates for visible and NIR electrochromic applications.

## 2 Experimental Section

### 2.1 Synthesis

Five D-π-A-π-D compounds, namely, 2,5-bis(5-(4-(2-ethylhexyl)-4*H*-dithieno[3,2-*b*:2′,3′-*d*]pyrrol-2-yl)thiophen-2-yl)-1,3,4-thiadiazole (**DTP-TD**), 4,7-bis(5-(4-(2-ethylhexyl)-4*H*-dithieno[3,2-*b*:2′,3′-*d*]pyrrol-2-yl)thiophen-2-yl)benzo[*c*][1,2,5]thiadiazole (**DTP-BTD**), 3,6-bis(5-(4-(2-ethylhexyl)-4*H*-dithieno[3,2-*b*:2′,3′-*d*]pyrrol-2-yl)thiophen-2-yl)-1,2,4,5-tetrazine (**DTP-TZ**), 2,5-bis(2-ethylhexyl)-3,6-bis(5-(4-(2-ethylhexyl)-4*H*-dithieno[3,2-*b*:2′,3′-*d*]pyrrol-2-yl)thiophen-2-yl)-2,5-dihydropyrrolo[3,4-*c*]pyrrole-1,4-dione (**DTP-DPP**), and 2,7-bis(2-ethylhexyl)-4,9-bis(5-(4-(2-ethylhexyl)-4*H*-dithieno[3,2-*b*:2′,3′-*d*]pyrrol-2-yl)thiophen-2-yl)benzo[*lmn*][3,8]phenanthroline-1,3,6,8(2*H*,7*H*)-tetraone (**DTP-NDI**) were obtained by Stille coupling (Scheme 1) in yields varying from 23% (**DTP-BTD**) to 71% (**DTP-TD**). The detailed preparation procedures are described in the Supporting Information, together with spectroscopic characterization and elemental analysis of the synthesized compounds.

### 2.2 Spectroscopic Studies

UV–vis absorption spectra of the obtained compounds in solution were recorded with a PerkinElmer Lambda 35 spectrophotometer. Fluorescence spectra were obtained using an FLS1000, Edinburgh Instruments. UV–vis absorption spectra of the compounds dispersed in the Zeonex matrix were recorded using a Hitachi UV-2300II spectrometer. Their emission spectra were recorded using an Edinburgh FS5 spectrofluorometer equipped with an enhanced range photomultiplier detector (PMT-EXT) using front face geometry. The measurements were performed at room temperature.

### 2.3 Electrochemical, Spectroelectrochemical, and Electrochromic Studies

Electrochemical (cyclic voltammetry (CV) and differential pulse voltammetry (DPV)) measurements were carried out using an Autolab potentiostat PGSTAT20 (Eco Chemie, the Netherlands). Cyclic voltammograms of the synthesized compounds, dissolved in 0.1 M Bu_4_NPF_6_ in a dichloromethane electrolyte, were typically registered at a scan rate of 50 mV/s and DPV curves at a modulation time of 50 ms, modulation amplitude of 10 mV, and step potential of 5 mV. The measurements were performed in an inert atmosphere using a platinum disk working electrode of a surface area of 2 mm^2^, a platinum wire counter electrode, and an Ag/0.1 M AgNO_3_/acetonitrile reference electrode, whose potential was verified at the end of each set of the experiment using the ferrocene (Fc/Fc^+^) couple. Thin polymer films deposited on the same platinum disk electrode, as described above, were studied in 0.1 M Bu_4_NPF_6_/CH_3_CN. For the investigation of spectroelectrochemical and electrochromic properties, polymer films were deposited on ITO electrodes by electrochemical polymerization of the corresponding monomers in a 0.1 M Bu_4_NPF_6_/CH_2_Cl_2_ electrolyte. They were then repeatedly washed with pure CH_2_Cl_2_ to remove soluble oligomers and transferred to the electrolyte consisting of 0.1 M Bu_4_NPF_6_ in CH_3_CN. In spectroelectrochemical measurements, UV–vis–NIR spectra were registered using a Varian Cary 5000 and a potentiostat μAUTOLAB type III. Electrochromic parameters such as CIE coordinates and transmittance plots were determined using a modular spectrometer StellarNet equipped with a reflectance probe, Ocean Optics QE65000 and NIRQuest512 diode array spectrometers, Autolab PGSTAT100N potentiostat, and Digital Camera Canon DS126271. The electrochromic measurements were carried out according to the procedure described previously.^38^

### 2.4 Quantum Chemical Calculations

Optimization of the ground-state molecular conformations of the neutral molecules was performed using hybrid Hartree–Fock/density functional theory (DFT) methods B3LYP^39,40^ and MN15.^41^ The B3LYP method usually provides accurate ground-state conformations and other basic properties of π-conjugated molecules, including their ion radicals.^42−44^ To account for the noncovalent interactions among conjugated rings, the B3LYP method was combined with the Grimme empirical dispersion correction (B3LYP-D3).^45^ The MN15 method is able to achieve very good accuracy in calculations of different kinds of molecular properties of a wide variety of compounds. Particularly, it has good accuracy simultaneously for valence, Rydberg, and charge-transfer electronic excitations, which is not achieved by most other functionals.^41^ This feature is very important for the determination of the correct order of excited states in compounds exhibiting both local and charge-transfer excitations. Open-shell species (ion radicals) were calculated by means of the spin-unrestricted approach. The Pople basis set 6-311+G* was used. As a standard procedure used in many quantum chemical studies, calculated molecules were simplified by replacing aliphatic substituents on nitrogens with methyl groups. Molecular conformations were calculated for both the isolated molecules (in vacuum) and molecules in dichloromethane (DCM), toluene, and dimethyl sulfoxide (DMSO) solutions. Solvation effects were described by means of the polarizable continuum model (PCM) using the integral equation formalism variant.^46^ The obtained equilibrium structures were checked by the normal mode analysis (no imaginary frequency was found).

Vertical and adiabatic ionization potentials and electron affinities were calculated as differences between the total energies of the neutral molecule and the corresponding ion radical (so-called the ΔSCF method). The vertical excited states (absorption spectra) were computed by the time-dependent version (TD)^47−49^ of the PCM-MN15/6-311+G* method at the ground-state molecular geometries using the linear response approach with the nonequilibrium solvation.^50^ To obtain the emission (fluorescence) spectra, excited-state S_1_ conformations were optimized by means of the same method but applying the Tamm–Dancoff approximation (TDA-PCM-MN15/6-311+G*) and assuming the equilibrium solvation. All calculations were performed using the Gaussian 16 program package.^51^

The exciton binding energy was determined as the difference of the vertical electrical and optical gaps calculated using the same DFT functional (either B3LYP-D3 or MN15).^52^ We suppose that the studied dissolved compounds create small aggregates, which in principle allow the separation of dissociated charges on adjacent molecules, but the solute concentration is so small that the dielectric constant of the solution may be approximated with the solvent dielectric constant. Thus, electrical and optical gaps were calculated for a single molecule surrounded by the solvent.

## 3 Results and Discussion

### 3.1 Spectroscopic Studies

**Figure 1:**
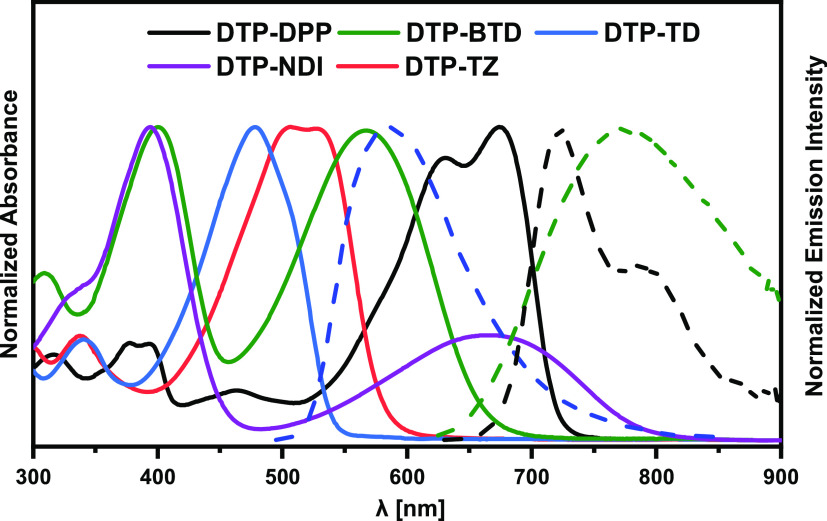
UV–vis–NIR absorption (solid lines) and emission intensity (dotted lines) spectra registered for DCM solutions of the studied compounds. Spectra were normalized to yield equal absorbance (emission intensity) for the most intensive band in the spectrum of each studied compound.

All D-π-A-π-D compounds shown in Scheme 1 consist of two dithienopyrrole donor units connected to the central acceptor units *via* two π-type linkers, namely, 2,5-thienylene groups, to yield D-π-A-π-D. In each particular case, the D-π units remain the same while the acceptor strengths of the central unit are varied. UV–vis spectra of their DCM solutions are shown in Figure 1, and the absorption maxima are collected in Table 1. None of the registered spectra can be considered as a simple superposition of the spectrum of dithieno[3,2-*b*:2′,3′-*d*]pyrrole with alkyl *N*-substituent and the corresponding spectra of compounds that mimic the π-A-π unit of the studied molecules, *i.e.*, thiadiazole, benzothiadiazole, diketopyrrolopyrrole, tetrazine, and naphthalene diimide disubstituted with thienyl groups. *N*-alkylated **DTP** gives rise to a strong band of vibronic character with a clear maximum in the vicinity of 300 nm corresponding to the dominant *0–1* transition.^53^ This band is either nonexistent or profoundly modified in the studied compounds. Moreover, in the spectra of the derivatives investigated in this research, bands of the lowest energy (longest wavelengths) dominate and are strongly bathochromically shifted with respect to the analogous bands in the spectra of compounds mimicking the π-A-π segments of the studied molecules, namely, 2,5-di(thiophen-2-yl)-1,3,4-thiadiazole,^54,55^ 4,7-di(thiophen-2-yl)benzo[*c*][1,2,5]thiadiazole,^56−58^ 3,6-di(thiophen-2-yl)-1,2,4,5-tetrazine,^59^ and 2,5-bis(2-ethylhexyl)-3,6-di(thiophen-2-yl)-2,5-dihydropyrrolo[3,4-*c*]pyrrole-1,4-dione.^60−62^

**Table 1:** Absorption and Emission Properties of the Studied Compounds Dissolved in DCM

compound	λ_max_^abs^ [nm]	λ_max_^em^ [nm]	ϕ [%]
**DTP-TD**	478, 340, 284	585	55
**DTP-BTD**	564, 400, 308	775	19
**DTP-TZ**	519, 338, 295		
**DTP-DPP**	672 (635), 461, 378, 318	722	19
**DTP-NDI**	661, 389		

Naphthalene diimides core-functionalized with donors of different strengths exhibit very characteristic UV–vis spectra. In this case, the lowest energy band (highest wavelength) corresponds to a band of CT character, whereas the band dominant in intensity is located at shorter wavelengths.^63,64^ In the spectrum of **DTP-NDI**, both the CT and dominant bands are strongly bathochromically shifted with respect to the corresponding bands in *N*,*N*′-bis(2-ethylhexyl)-2,6-dithiophene-1,4,5,8-naphthalene diimide, *i.e.*, molecule mimicking the π-A-π segment of **DTP-NDI**.^65^ Thus, by comparing the UV–vis–NIR spectra of D-π-A-π-D compounds studied in this research with the corresponding spectra of the compounds that mimic their central π-A-π segment, it can be concluded that the observed bathochromic shift of the least energetic band in the former is caused by two factors: extension of the π-system and donor–acceptor interactions. Stronger D-A interactions induce a larger bathochromic shift. It increases with increasing acceptor strength, being 66 nm for **DTP-TD**, 104 nm for **DTP-BTD**, 120 nm for **DTP-DPP**, and 187 nm for **DTP-NDI**. The UV–vis spectra of the studied compounds are weakly dependent on the solvent polarity. However, the dominant band is shifted by *ca.* 15–18 nm when a nonpolar solvent (hexane) is replaced with a highly polar one, *e.g.*, acetonitrile or DMSO (Table S1).

Out of the five compounds studied, three are luminescent (**DTP-TD**, **DTP-BTD**, and **DTP-DPP**). Their representative photoluminescence spectra recorded for dichloromethane solutions are presented in Figure 1. Emission spectra of the three luminescent compounds are much more solvent-sensitive than their UV–vis ones. In particular, with increasing solvent polarity, the Stokes shift increases, while the photoluminescence quantum yield (PLQY) strongly decreases (Table S1). For example, in the case of the most luminescent **DTP-TD**, λ_max_^em^ shifts from 543 nm in toluene solutions to 635 nm in DMSO solutions, whereas the PLQY decreases from 64 to 36%. This effect is even more pronounced for **DTP-BTD**, where the λ_max_^em^ shifts from 713 nm to 860 nm and the PLQY decreases from 50% to 2.6% when toluene is replaced with DMSO. Spectroscopic investigations carried out for solutions of these two luminophores in a variety of solvents show clear correlations between the PLQY (or λ_max_^em^) and the relative polarities of the solvents. Selected photophysical properties of the luminescent compounds are presented in Table 1, whereas the remaining spectroscopic data obtained for different solvents can be found in Table S1 in the Supporting Information. These large Stokes shifts, which additionally increase with increasing solvent polarities, indicate that the geometries of the molecules in their excited state significantly differ from those of the ground state, giving rise to large and solvent-dependent differences in the corresponding dipole moments.

**Figure 2:**
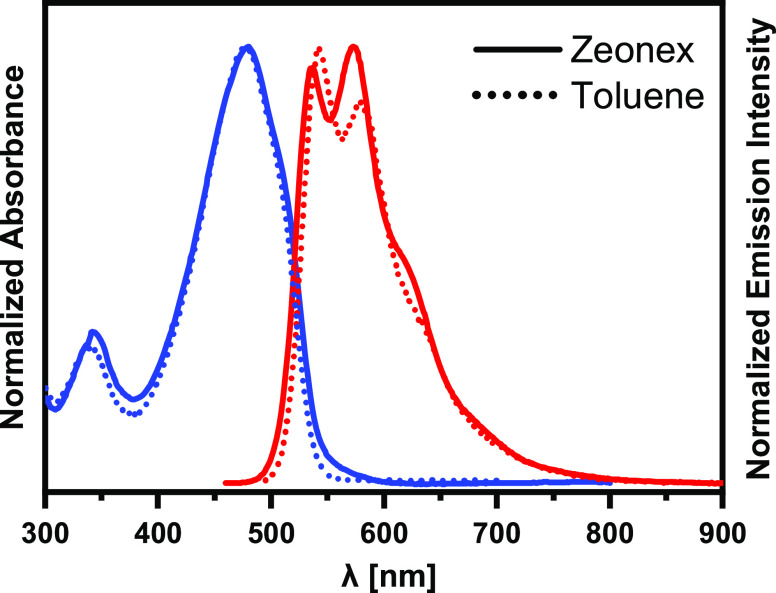
Absorption and photoluminescence spectra registered for the molecular dispersion of **DTP-TD** in Zeonex (0.3%) (solid lines) and **DTP-TD** in toluene solution (dotted lines). Spectra were normalized to yield equal absorbance and emission intensity of the most intensive bands of **DTP-TD** solutions in toluene and dispersions in Zeonex.

Potential applications of these newly elaborated luminophores require detailed characterization of their luminescent properties in the solid-state, for example, by molecularly dispersing them in appropriate matrices, usually of polymeric nature. The Zeonex matrix is very well suited for this purpose since the resulting layers are uniform and yield spectra of good quality. UV–vis–NIR spectra of **DTP-TD** dispersed in Zeonex are concentration-independent, at least in the studied concentration range 0.05–5%, see Figure S1 in the Supporting Information. Representative absorption and emission spectra of the dispersion of **DTP-TD** (0.3%) are shown in Figure 2. It should be noted that the absorption spectrum of this compound closely resembles that registered for its solution in toluene. Moreover, λ_max_^em^ values in the Zeonex matrix (536 nm) almost coincide with the corresponding value of the spectrum registered in toluene (543 nm). Taking into account the close similarity of dielectric constants of Zeonex and toluene, it can be concluded that the resemblance of the solution and dispersion spectra manifests similar interactions of the solvent and the matrix with the luminophore molecules. As a consequence, it implies truly molecular dispersions of **DTP-TD** in Zeonex, at least for lower concentrations of the luminophore. Vibrational structures, albeit of different intensity sequences, are clearly visible in both emission spectra. In the case of the dispersion in Zeonex (Figure S2), this band of vibrational nature can be deconvoluted into three components whose relative intensities change with the increasing concentration of the luminophore.

To summarize this part of the paper, we have demonstrated that by varying DA interactions in the studied D-π-A-π-D compounds, it is possible to precisely tune their spectroscopic properties in terms of absorption and emission. This applies not only to solution spectra but also to spectra recorded for dispersions of the studied luminophores in Zeonex. A detailed explanation of the experimentally observed spectroscopic phenomena, however, requires theoretical support. The results of the theoretical calculations are summarized in the subsection devoted to DFT calculations (*vide infra*).

### 3.2 Electrochemical Characterization

**Figure 3:**
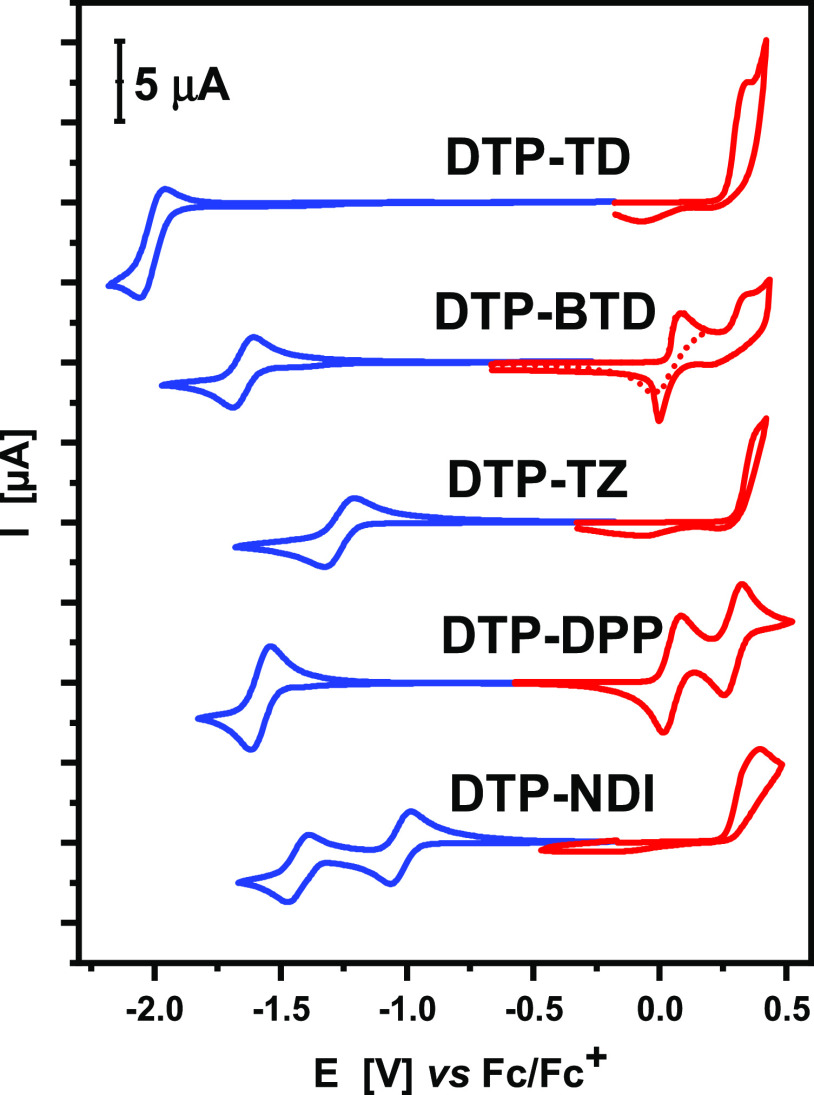
Cyclic voltammograms of **DTP-TD, DTP-BTD**, **DTP-TZ**, **DTP-DPP**, and **DTP-NDI;** concentration, 10^–3^ M; electrolyte, 0.1 M Bu_4_NPF_6_/CH_2_Cl_2_; scan rate, 50 mV/s.

The determination of redox properties of new electroactive compounds is of crucial importance as far as their electronic or electrochromic applications are considered. D-π-A-π-D compounds frequently show ambipolarity^63^ since their donor (electron-rich) parts are easy to oxidize, whereas the acceptor (electron-poor) parts are easy to reduce. As a result, low IP and high |EA| values are expected, leading to low electrochemical band gaps. This is also the case with the compounds described in this research; however, their electrochemical behavior differs as probed by cyclic voltammetry (Figure 3) and differential pulse voltammetry (Figure S3).

**DTP-DPP** distinguishes itself from the other four investigated compounds since it does not oxidatively electropolymerize. Instead, it undergoes a two-step reversible oxidation at potentials of 0.05 and 0.29 V *vs* Fc/Fc^+^ (see Figure 3). A similar two-step oxidation was reported for DPP-centered D-π-A-π-D compounds with weaker donors, albeit at significantly higher potentials.^60,61^ This two-step process can be ascribed to the consecutive oxidations of the neutral molecule to a radical cation and spinless dication, similar to that demonstrated by EPR spectroelectrochemistry for very similar compounds consisting of the same diketopyrrolopyrrole (**DPP**) acceptor and thiophene, bithiophene, and terthiophene donors.^60^ High stabilities of the radical cation and dication forms prevent **DTP-DPP** from its oxidative electropolymerization. At negative potentials, it undergoes a reversible reduction to a radical anion form. It should be noted that the potential of this reduction is 100 mV higher as compared to the reduction of 2,5-bis(2-ethylhexyl)-3,6-di(thiophen-2-yl)-2,5-dihydropyrrolo[3,4-*c*]pyrrole-1,4-dione, *i.e.*, compound that mimics the π-A-π part of **DTP-DPP**.^61^ This can be rationalized by joint effects of extended conjugation and donor–acceptor interactions.

Electropolymerization of D-π-A-π-D compounds is usually initiated by radical cation generation at terminal groups of the molecule, followed by recombination of the formed radicals with the simultaneous abstraction of protons. From this perspective, the electrochemical behavior of **DTP-BTD** is not typical. At the potential of its first oxidation, no electropolymerization occurs. Thus, when the vertex potential is limited to +0.2 V *vs* Fc/Fc^+^, a partly reversible redox couple is registered (see Figure 3). This means that the electropolymerization cannot be initiated by the presence of radical cations in this case. However, if the vertex potential is extended over the second oxidation process, *i.e.*, to +0.425 V *vs* Fc/Fc^+^, quick electropolymerization takes place. Thus, **DTP-BTD** starts to electropolymerize only if its spinless dicationic state is reached. C–C coupling of **DTP-BTD** molecules must therefore proceed *via* proton abstraction from the formed dications, followed by recombination of the resulting free radicals. At negative potentials (*E* = −1.65 V *vs* Fc/Fc^+^) **DTP-BTD** undergoes a reversible reduction to a radical anion. Again, this potential is *ca.* 100 mV higher than the corresponding reduction potential of 4,7-di(thiophen-2-yl)benzo[*c*][1,2,5]thiadiazole, *i.e.*, the compound mimicking the π-A-π segment of **DTP-BTD**.^56,57,66^ The remaining three compounds readily electropolymerize at relatively low potentials. The separation of their polymerization process from its initiation (*i.e.*, radical cation generation) by CV or DPV investigations is impossible in these cases. **DTP-NDI**, **DTP-TZ**, and **DTP-TD** start to electropolymerize at *E*_onset_ values of 0.26, 0.30, and 0.26 V *vs* Fc/Fc^+^, respectively (see Figure 3). All three compounds are different from **DTP-BTD** and **DTP-DPP** in the sense that their reduction potentials are slightly influenced by D-A interactions and are similar to the corresponding potentials of compounds mimicking their π-A-π segments, *i.e*., *N,N*′-bis(2-alkyl)-2,6-dithiophene-1,4,5,8-naphthalene diimide,^65^ 3,6-di(thiophen-2-yl)-1,2,4,5-tetrazine,^59^ and 2,5-di(2-thienyl)-1,3,4-thiadiazole.^55^ In particular, **DTP-NDI** undergoes a reversible two-step reduction to a radical anion in the first step (*E*^0/–1^ = −1.03 V *vs* Fc/Fc^+^) and to a spinless dianion in the second step (*E*^–1/–2^ = −1.43 V *vs* Fc/Fc^+^), as previously reported for naphthalene diimides core-functionalized with other donors.^64^ In the cyclic voltammogram of **DTP-TZ**, one reduction process of quasireversible nature can be distinguished with the reduction peak at *E*^0/–1^= −1.28 V *vs* Fc/Fc^+^. **DTP-TD** is the only compound of the studied series whose reduction is irreversible. This is associated with a significantly lower potential of its reduction process, which starts at *E*_onset_= −1.94 V *vs* Fc/Fc^+^, rendering the formed radical anion unstable and prone to subsequent degradation reactions of irreversible nature.

From the electrochemical data presented above, it is possible to determine the ionization potential and electron affinity of the studied compounds. In principle, IP should be calculated using the formal redox potential of the first oxidation process (*E*^0ox1^ = 1/2(*E*^0/+1^ + *E*^+1/0^)), whereas EA should be calculated from the formal potential of the first reduction process (*E*^0red1^ = 1/2(*E*^0/-1^ + E^–1/0^)). This procedure can be applied to **DTP-DPP** and **DTP-BTD** only. In the remaining three cases, the redox process corresponding to the first oxidation is totally obscured by the electropolymerization phenomenon, which is irreversible in nature. Additionally, the reduction peak registered for **DTP-TD** is also irreversible. Therefore, for comparative reasons, we used the potentials of the onsets of the first oxidation and the first reduction peaks in our calculations of IPs and EAs. This is a common procedure in the treatment of electrochemical data of compounds exhibiting the irreversibility of their redox processes. The IP and EA values obtained from electrochemical measurements are listed in Table 2. Significant differences between the EA values of particular compounds should be noted, the largest difference approaching 1 eV being observed between **DTP-NDI** and **DTP-TD**. The range of IP values is smaller; however, the IP values of **DTP-DPP** and **DTP-BTD** are lower by 0.23–0.31 eV than IPs of the remaining three compounds.

**Table 2:** Ionization Potentials (IP_el_) and Electron Affinities (EA_el_) of the Synthesized Compounds, Derived from Cyclic Voltammetry Results

compound	*E*_onset_^ox^ [V]	*E*_onset_^red^ [V]	IP_el_^a^ [eV]	EA_el_^a^ [eV]	Eg_el_ [eV]
**DTP-TD**	0.26	–1.94	5.06	–2.86	2.20
**DTP-BTD**	0.03	–1.57	4.83	–3.23	1.60
**DTP-TZ**	0.30	–1.20	5.10	–3.60	1.50
**DTP-DPP**	–0.01	–1.51	4.79	–3.29	1.50
**DTP-NDI**	0.26	–0.96	5.06	–3.84	1.22

a Determined using the following relationship: IP[eV] = |*e*|(*E*_onset_^ox^ + 4.8) eV and EA[eV] = −|*e*|(*E*_onset_^red^ + 4.8) eV.

### 3.3 Quantum Chemical Calculations

Detailed elucidation of the spectroscopic and electrochemical data presented in the two previous subsections requires theoretical support. For this purpose, DFT calculations were performed using two different DFT functionals (B3LYP-D3 and MN15) with the goal to improve the reliability of the results. In particular, molecular conformations of neutral molecules as well as their radical anion and cation forms both in vacuum and DCM solutions were optimized. Both applied methods yielded very similar ground-state equilibrium conformations. Dihedral angles between the donor and the π-bridge and between the π-bridge and the acceptor in the neutral molecules, obtained using the B3LYP-D3 method, are listed in Table 3. While **DTP-TD**, **DTP-BTD**, **DTP-TZ**, and **DTP-DPP** derivatives are basically planar with dihedral angles between neighboring moieties inferior to 20°, π-bridges in **DTP-NDI** are significantly twisted with respect to the acceptor part of the molecule. The lowest-energy conformers of **DTP-TD**, **DTP-BTD**, and **DTP-NDI** are of *C*_2_ symmetry, whereas **DTP-TZ** and **DTP-DPP** exhibit *C*_*i*_ symmetry both in vacuum and in DCM. These symmetries are, however, broken in some radical ions in DCM solution, as shown in Table S2 of the Supporting Information. Analogous results obtained using the MN15 method are presented in Table S3. Unlike B3LYP, MN15 predicts a small *C*_*i*_ symmetry breaking also in the neutral form of **DTP-DPP**.

**Table 3:** Dihedral Angles (in Degrees) between the Donor−π Bridge and π Bridge–Acceptor Parts of the Neutral Molecule Optimized Using the (PCM-)B3LYP-D3/6-311+G* Method in Vacuum and DCM Solution

solvent/compound	donor−π bridge	π bridge–acceptor	acceptor−π bridge	π bridge–donor	symmetry
Vacuum					
**DTP-TD**	18.6	1.6	1.6	18.6	*C*_2_
**DTP-BTD**	18.3	9.1	9.1	18.3	*C*_2_
**DTP-TZ**	16.5	0.8	0.8	16.5	*C*_*i*_
**DTP-DPP**	15.9	0.6	0.6	15.9	*C*_*i*_
**DTP-NDI**	24.4	48.9	48.9	24.4	*C*_2_
DCM					
**DTP-TD**	10.4	2.4	2.4	10.4	*C*_2_
**DTP-BTD**	16.1	13.2	13.2	16.1	*C*_2_
**DTP-TZ**	11.9	0.6	0.6	11.9	*C*_*i*_
**DTP-DPP**	12.2	0.6	0.6	12.2	*C*_*i*_
**DTP-NDI**	21.0	48.6	48.6	21.0	*C*_2_

Table 4 shows the two lowest vertical excited states S_1_ and S_2_ (absorption) and the lowest relaxed excited states S_1_ (luminescence) obtained using the MN15 functional. For molecules in DCM solution, the calculated vertical excitation energies exhibiting noticeable oscillator strengths well coincide with the experimental absorption spectra. Excitation energies of the relaxed S_1_ states in **DTP-TD** and **DTP-DPP** are characterized by significant oscillator strengths, which agrees with their experimentally observed fluorescence. Contrary to other studied compounds, it was found that the observed lowest energy absorption peak of **DTP-TZ** corresponds to the second excited state S_2_. Thus, after excitation, this molecule undergoes vibrational relaxation to the S_1_ state (Kasha’s rule). Since the oscillator strength of the S_1_ → S_0_ transition is negligible, the fluorescence of this compound is not possible. In the case of **DTP-NDI** absorption to its lowest vertical excited state, S_1_ takes place with a rather high oscillator strength. However, after vibrational relaxation of this state, which is connected to an increase of the dihedral angle between one of the π-donor moieties and the acceptor central unit from *ca.* 49° (see Tables S2 and S3 in the Supporting Information) to *ca.* 89° (see Table S4 in the Supporting Information), the oscillator strength of the S_1_ → S_0_ transition drops almost to zero. Consequently, the fluorescence of the **DTP-NDI** compound is not observed experimentally.

**Table 4:** Wavelengths λ and Oscillator Strengths *f* of Two Lowest Vertical Excited States S_1_ and S_2_ (Absorption Peaks) and the Lowest Relaxed Excited States S_1_ (Luminescence Peak) of the Studied Compounds in Vacuum and DCM Solution Obtained Using the TD (PCM-)MN15/6-311+G* Method^a^

	absorption peaks				luminescence	
	S_1_		S_2_		S_1_	
solvent/compound	λ [nm]	*f*	λ [nm]	*f*	λ [nm]	*f*
Vacuum						
**DTP-TD**	455	2.17	387	0.07	523	2.87
**DTP-BTD**	561	1.28	436	0.11	649	1.65
**DTP-TZ**	587	0.00	478	2.34	642	0.01
**DTP-DPP**	590	1.99	421	0.00	613	3.27
**DTP-NDI**	629	0.66	574	0.04	1104	0.00
DCM						
**DTP-TD**	482	2.37	413	0.08	604	2.87
**DTP-BTD**	566	1.47	443	0.16	759	1.94
**DTP-TZ**	579	0.00	510	2.52	628	0.01
**DTP-DPP**	639	2.24	453	0.00	769	3.02
**DTP-NDI**	640	0.73	584	0.05	1003	0.00

a Tamm–Dancoff approximation was used for the calculation of luminescence.

Light absorption in organic materials usually results in the formation of excitons (mostly Frenkel-type excitons) rather than in the formation of free charge carriers as in inorganic semiconductors. Excitons are stabilized by Coulomb interactions, frequently referred to as exciton binding energy. If the exciton binding energy is comparable to or smaller than the thermal energy kT, excitons may dissociate into free charge carriers instead of undergoing radiative deexcitation (fluorescence).^26^ These processes can be expected not only in the solid-state samples but also in nanoaggregates. Thus, to elucidate the effect of the solvent dielectric constant on the luminescence quantum yield of **DTP-TD** and **DTP-BTD**, the exciton binding energies in vacuum and in different solvents were calculated (see Table 5). The data obtained for these compounds unequivocally indicate that the exciton binding energy strongly decreases with increasing solvent dielectric constant, dropping to a very small value for DMSO, the most polar of all solvents investigated. Consequently, the luminescence quantum yield is expected to decrease with an increase in the solvent dielectric constant. This was observed experimentally. For solutions in the same solvents, exciton binding energies of **DTP-TD** were found to be slightly greater than those calculated for **DTP-BTD**. It is in good agreement with the measured quantum yields of photoluminescence, being higher for **DTP-TD** than for **DTP-BTD** but showing similar quenching in highly polar solvents.

**Table 5:** Exciton Binding Energies of DTP-BTD and DTP-TD Dissolved in Different Solvents

compound/solvent	exciton binding energy [eV]	
method:	(PCM-)B3LYP/6-311+G*	(PCM-)MN15/6-311+G*
**DTP-BTD**		
vacuum	2.22	2.27
toluene	0.99	0.98
DCM	0.28	0.24
DMSO	0.05	0.002
**DTP-TD**		
vacuum	2.21	2.40
toluene	0.99	1.14
DCM	0.29	0.42
DMSO	0.08	0.20

Theoretical calculations also provide important information concerning the redox properties of the studied compounds. More specifically, they help to elucidate their oxidation to the radical cation state and their reduction to the radical anion form. Table 6 shows Mulliken spin densities obtained from the population analysis of the ground-state electronic densities of the radical ions in vacuum and in DCM solution summed on particular groups. Isosurfaces of these spin densities are depicted in Figures S4 and S5 of the Supporting Information. The occurrence of some smaller areas with negative values results from the use of the spin-unrestricted wave function. It is obvious that the unpaired electron in the radical cation formed through oxidation of **DTP-NDI** is predominantly localized in its donor−π parts. This localization is supported by a significant twist between the π-bridge and the acceptor part, which breaks the conjugation. Note that the asymmetric localization of the unpaired electron in the radical cation form of **DTP-NDI** in DCM corresponds to the broken symmetry of the molecular conformation (see Table S2 in the Supporting Information). By analogy, the unpaired electron in the radical anion form of **DTP-NDI** is located in its acceptor part. On the other hand, unpaired electrons in the radical-ion forms of **DTP-TD** and **DTP-DPP** are delocalized over the whole molecules, which is related to their nearly planar conformations, proving strong coupling between the donor and the acceptor parts. It should, however, be noted that unpaired electrons are also localized in the radical-ion forms of **DTP-BTD** and **DTP-TZ** despite their highly planar shape. This can be tentatively ascribed to the strong acceptor character of the central unit.

**Table 6:** Mulliken Spin Densities of Radical Ions of the Studied Derivatives in Vacuum and DCM Solution Calculated by the (PCM-)B3LYP-D3/6-311+G* Method^a^

solvent/compound	charge	donor	π-bridge	acceptor	π-bridge	donor
Vacuum						
**DTP-TD**	cation	0.304	0.154	0.083	0.154	0.304
	anion	0.163	0.209	0.256	0.209	0.163
**DTP-BTD**	cation	0.273	0.177	0.099	0.177	0.273
	anion	0.092	0.101	0.612	0.101	0.092
**DTP-TZ**	cation	0.335	0.165	0.002	0.165	0.335
	anion	0.151	0.178	0.341	0.178	0.151
**DTP-DPP**	cation	0.194	0.126	0.360	0.126	0.194
	anion	0.148	0.153	0.398	0.153	0.148
**DTP-NDI**	cation	0.347	0.148	0.010	0.148	0.347
	anion	0.011	0.019	0.939	0.019	0.011
DCM						
**DTP-TD**	cation	0.309	0.153	0.077	0.153	0.309
	anion	0.128	0.215	0.313	0.215	0.128
**DTP-BTD**	cation	0.269	0.179	0.103	0.179	0.269
	anion	0.053	0.084	0.725	0.085	0.053
**DTP-TZ**	cation	0.344	0.159	-0.007	0.159	0.345
	anion	-0.008	0.007	1.001	0.007	-0.008
**DTP-DPP**	cation	0.199	0.132	0.337	0.132	0.199
	anion	0.115	0.149	0.471	0.149	0.115
**DTP-NDI**	cation	0.016	0.010	0.021	0.263	0.691
	anion	0.008	0.018	0.948	0.018	0.008

a Spin densities are summed on the donor, π-bridge, and acceptor groups.

Table 7 shows frontier orbital energies (HOMO, LUMO) together with vertical and adiabatic ionization potentials and electron affinities calculated using the B3LYP-D3 method and theoretical band gaps obtained as a difference between the IP and the negative value of EA. The calculated adiabatic IPs and EAs of the studied compounds in DCM solution are in excellent agreement with experimental values obtained from the electrochemical measurements (compare data in Tables 2 and 7).

**Table 7:** Frontier Orbital Energies (HOMO, LUMO), Vertical and Adiabatic Ionization Potentials (IP) and Electron Affinities (EA), and Theoretical Band Gaps (Eg) Calculated in Vacuum and in DCM Solution by Means of the (PCM-)B3LYP-D3/6-311+G* Method

			vertical			adiabatic		
solvent/compound	HOMO [eV]	LUMO [eV]	IP [eV]	EA [eV]	Eg [eV]	IP [eV]	EA [eV]	Eg [eV]
Vacuum								
**DTP-TD**	–5.12	–2.54	6.03	–1.56	4.48	5.90	–1.67	4.23
**DTP-BTD**	–4.92	–2.92	5.81	–1.87	3.94	5.66	–1.98	3.68
**DTP-TZ**	–5.16	–2.76	6.07	–1.75	4.32	5.95	–1.84	4.11
**DTP-DPP**	–4.80	–2.87	5.71	–1.95	3.76	5.54	–2.02	3.52
**DTP-NDI**	–5.22	–3.57	6.07	–2.53	3.54	5.96	–2.66	3.30
DCM								
**DTP-TD**	–5.18	–2.69	5.15	–2.75	2.41	5.05	–2.84	2.21
**DTP-BTD**	–5.01	–3.01	4.97	–3.04	1.93	4.83	–3.15	1.68
**DTP-TZ**	–5.21	–3.01	5.19	–3.00	2.18	5.08	–3.33	1.75
**DTP-DPP**	–4.93	–3.04	4.89	–3.11	1.78	4.75	–3.17	1.58
**DTP-NDI**	–5.25	–3.64	5.23	–3.67	1.56	5.10	–3.81	1.30

In summary, it has to be stated that the predictive values of the theoretical calculations presented in this research are excellent since they correctly estimate the spectroscopic properties of the investigated compounds. This includes, in particular, their absorption and emission spectra as well as the strong dependence of their photoluminescence quantum yields (PLQYs) on solvent polarity. Moreover, the calculated values of adiabatic ionization potentials and electron affinities are in excellent agreement with the corresponding data derived from the electrochemical measurements. This applies not only to the trends but also to the extremely close similarity of theoretical and experimental values.

### 3.4 Electropolymerization and Spectroelectrochemistry

As stated in Section 3.2, out of the five compounds studied, **DTP-DPP** does not electropolymerize. In the case of the remaining four compounds, uniform polymer films can be deposited on the working electrode through voltammetric electropolymerization. Representative electropolymerization scans are shown in Figure S6 of the Supporting Information.

**Figure 4:**
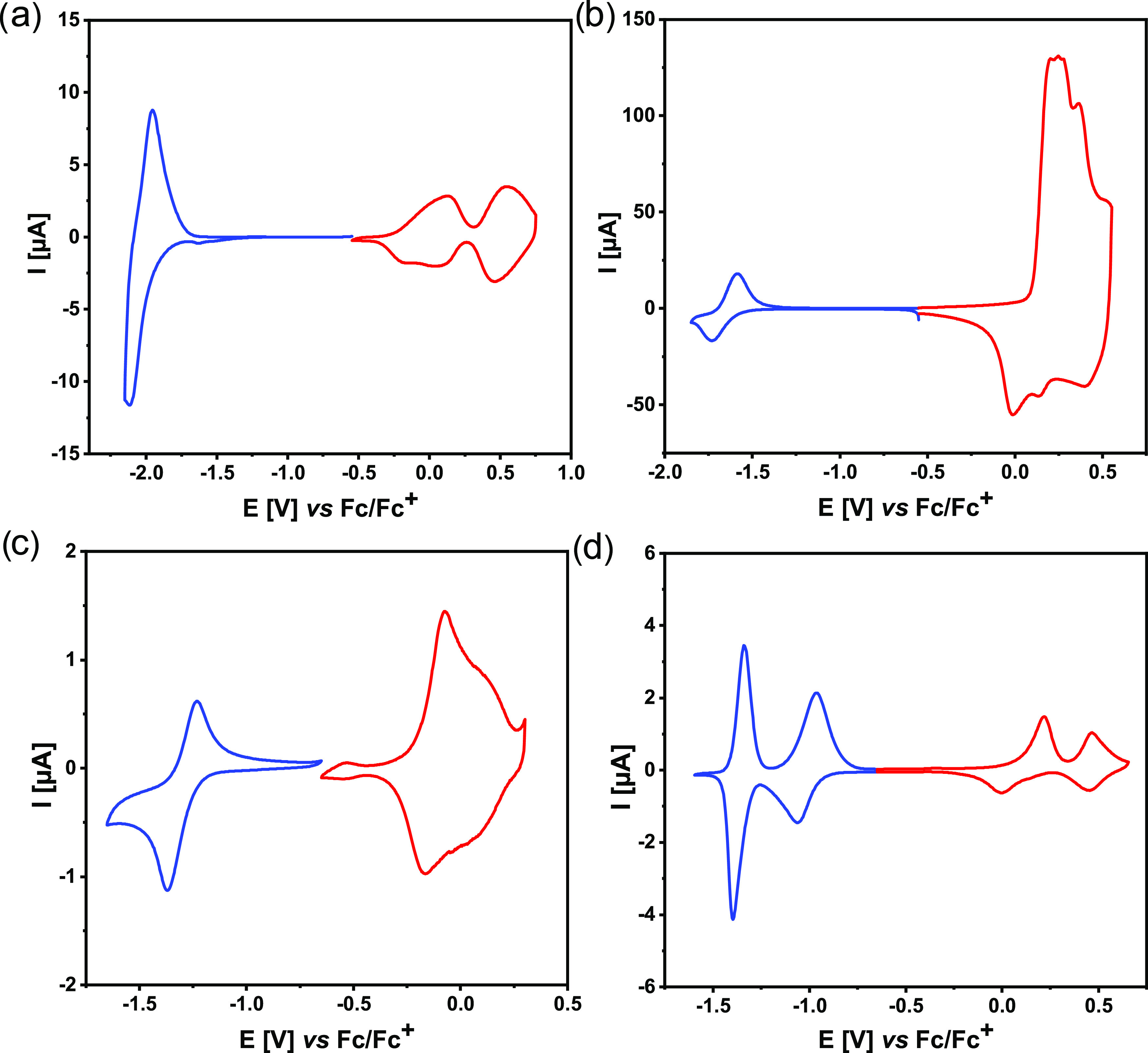
Cyclic voltammograms of **poly(DTP-TD)** (a), **poly(DTP-BTD)** (b), **poly(DTP-TZ)** (c), and **poly(DTP-NDI)** (d); electrolyte, 0.1 M Bu_4_NPF_6_/CH_3_CN; scan rate, 50 mV/s.

Cyclic voltammograms of the electropolymerization products are compared in Figure 4. All films deposited electrochemically on a platinum electrode can be electrochemically oxidized and reduced, albeit their redox potentials significantly differ, depending on the electron-accepting properties of their central unit. Note that poly(DTP-BTD) shows a much stronger electrochemical activity in the oxidation mode than in the reduction mode (see Figure 4b). This is manifested by significantly higher currents recorded for the polymer oxidation peaks at positive potentials as compared to those corresponding to its reduction at negative potentials. Redox potentials, together with IP and EA values calculated on the basis of these electrochemical data, are listed in Table 8.

**Table 8:** Ionization Potentials (IP_el_) and Electron Affinities (EA_el_) of the Studied Electropolymerization Products, Derived from Cyclic Voltammetry Results

polymer	*E*_onset_^ox^ [V]	*E*_onset_^red^ [V]	IP_el_^a^ [eV]	EA_el_^a^ [eV]	Eg^el^ [eV]	Eg^opt^ [eV]
**poly(DTP-TD)**	–0.25	–1.90	4.55	–2.90	1.65	1.71
**poly(DTP-BTD)**	0.11	–1.57	4.91	–3.23	1.68	1.36
**poly(DTP-TZ)**	–0.21	–1.23	4.59	–3.57	1.02	1.55
**poly(DTP-NDI)**	0.09	–0.94	4.89	–3.86	1.04	0.84

a Determined using the following relationship: IP[eV] = |e|(*E*_onset_^ox^ + 4.8) eV and EA[eV] = −|e|(E_onset_^red^ + 4.8)eV.

In the case of **poly(DTP-TD)** (Figure 4a), oxidation is quasireversible and corresponds to consecutive oxidations of two dithienopyrrole units yielding, respectively, the radical cation and dication forms of the oxidized polymer. Cyclic voltammograms of **poly(DTP-TD)** (Figure 4a), **poly(DTP-BTD)** (Figure 4b), and **poly(DTP-TZ)** (Figure 4c) registered at positive potentials resemble typical voltammograms resulting from the oxidative doping of conjugated polymers with a significant contribution of the capacitive current.^67^ Reduction of **poly(DTP-TD)** starts at lower potentials than in other polymers studied in this research, which manifests the weakest electron-accepting properties of the thiadiazole central unit. **Poly(DTP-NDI)** is especially interesting, showing five quasireversible oxidation states (see Figure 4d).

With the exception of **poly(DTP-BTD)**, the electropolymerization products show significantly lower electrochemical band gaps (Eg^el^) than the corresponding monomers (compare data presented in Table 2 and Table 8). This electropolymerization-induced band gap narrowing is caused by a significant decrease of IPs of the resulting polymers, while their EAs remain affected only slightly. From these data, it follows that the studied compounds in their oxidation processes behave like typical conducting polymers whose oxidation potential depends on the conjugation length and the facility to delocalize the positive charge. Finally, the strong ambipolarity of **poly(DTP-NDI)** and **poly(DTP-TZ)**, leading to low band gaps, should be pointed out.

The determined optical band gaps (Eg^opt^) of **poly(DTP-BTD)** and **poly(DTP-NDI)** are lower than the electrochemical band gaps (Eg^el^, see Table 8). This is a general phenomenon in these low and high molecular mass electroactive compounds, for which the HOMO to LUMO transitions can be probed spectroscopically.^68^ In these cases, (Eg^el^ – Eg^opt^) can be considered as the effective exciton binding energy.^26^ However, the Eg^opt^ of **poly(DTP-TZ)** is significantly higher than its Eg^el^. This apparent discrepancy is caused by the fact that in this compound, the HOMO to LUMO transition exhibits negligible oscillator strength and cannot be optically detected,^59^ whereas the cyclic voltammetry probes the energy of the HOMO as well as the energy of the LUMO. This is a general phenomenon in the case of aryl derivatives of tetrazines as reported in numerous previous papers^59,69^ and later ones.^70,71^

**Figure 5:**
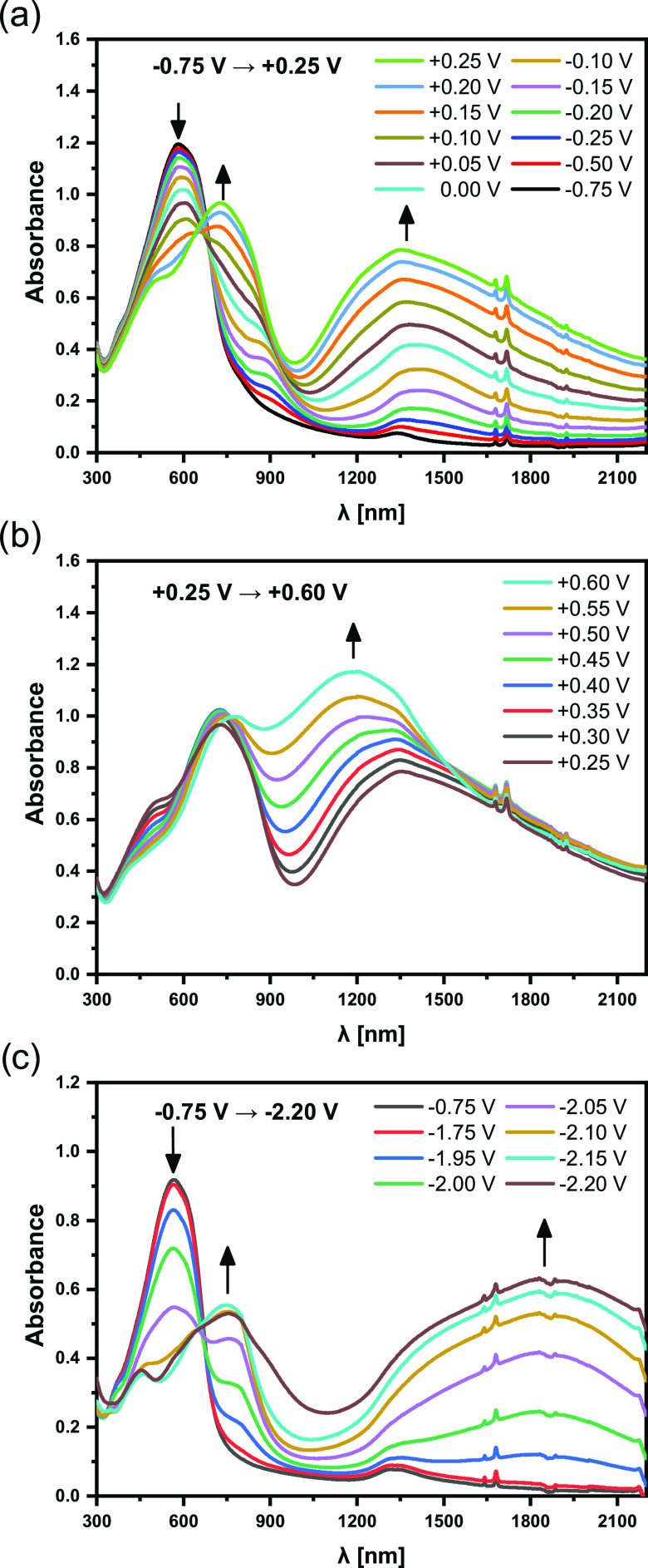
UV–vis–NIR spectra of a thin layer of **poly(DTP-TD**) deposited on ITO, recorded for increasing electrode potential from −0.75 to +0.25 V (a), from +0.25 to +0.60 V (b), and decreasing electrode potential from −0.75 to −2.20 V (c); electrolyte, 0.1 M Bu_4_PF_6_/CH_3_CN; potentials *vs* Fc/Fc^+^.

For UV–vis–NIR spectroelectrochemical investigations, thin polymer layers were deposited on ITO using voltammetric electropolymerization. The results of the spectroelectrochemical experiments carried out for **poly(DTP-TD)** are presented in Figure 5. At *E* = −0.75 V, the polymer is in its neutral state. Spectral features characteristic of the oxidized (doped) state start to appear at *E* = −0.25 V, coinciding with the onset of the first oxidation peak in the cyclic voltammogram (compare Figures 4a and 5a). The observed spectral changes are typical of oxidative doping of conducting polymers rather than oxidation processes occurring in redox-type polymers. The spectroelectrochemical data are presented in Figure 5a,b as two sets of spectra recorded in the potential ranges corresponding to the first and the second oxidation process, respectively. The first oxidation process can be briefly characterized as follows: with increasing electrode potential, the least energetic band of **poly(DTP-TD)** at 581 nm, characteristic of its neutral state, gradually bleaches. Concomitantly, two new peaks appear in the NIR region of the spectrum and grow in intensity with increasing potentials. At *E* = +0.25 V, *i.e.*, at the potential of the end of the first oxidation process, these oxidative doping-induced peaks are located at 1354 and 731 nm. In the second oxidation process, the band at 1354 nm undergoes increasing hypsochromic shift with increasing potential, being located at 1189 nm at *E* = +0.60 V. The band at 731 nm remains essentially unchanged. These new bands can be ascribed to radical cationic and dicationic forms of the polymer chain, *i.e.*, in terms of the solid-state physics to positive polarons and bipolarons.^72^ Thus, electrochemically oxidized **poly(DTP-TD**) shows spectroscopic features of a conducting polymer, albeit of moderate conductivity, as the presence of two clearly distinguishable doping-induced NIR peaks strongly suggests localization of doping-generated polarons and bipolarons.^72^

The spectral response of **poly(DTP-TD**) upon its electrochemical reduction is somehow similar (Figure 5c). Increasing the negative polarization of the electrode results in the appearance of two reduction-induced bands at *E* = −1.7 V *vs* Fc/Fc^+^ and their further growth with a concomitant decrease of the band ascribed to the neutral state of the polymer. At *E* = −2.2 V, the reduction-induced bands are located at 1826 and 754 nm. They can be ascribed to the presence of radical anions and dianions or in terms of solid-state physics to negative polarons and bipolarons.^72^ Thus, as probed by spectroelectrochemical techniques, **poly(DTP-TD**) behaves like a typical ambipolar conducting polymer. Since the reductive doping of this polymer requires polarizations to very low potentials, its stability upon cycling is limited, and appropriate spectroscopic data can be obtained upon the first cycle only.

**Figure 6:**
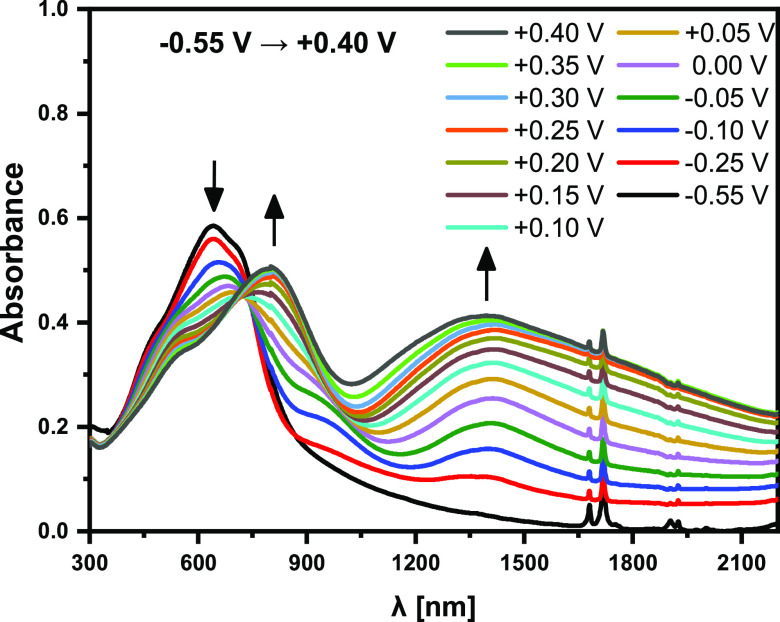
UV–vis–NIR spectra of a thin layer of **poly(DTP-TZ**) deposited on ITO, recorded for increasing electrode potential in the potential ranges from −0.55 to +0.40 V; electrolyte, 0.1 M Bu_4_PF_6_/CH_3_CN; potentials *vs* Fc/Fc^+^.

The oxidation potential of **poly(DTP-TZ)** and, by consequence, its IP is similar to those determined for **poly(DTP-TD**). Its reduction potential is, however, higher by nearly 670 mV, leading to a much higher |EA| (see Table 8). Despite the relatively high reduction potential of this polymer, its anionic forms are not very stable, and the results of its spectroelectrochemical investigations at negative potentials are not fully reproducible. On the contrary, its electrochemical oxidation leads to a clear and reproducible spectral response. As seen in Figure 6, the spectroelectrochemical behavior of **poly(DTP-TZ)** is also typical of conducting polymers and consistent with its cyclic voltammogram. Its oxidative doping starts at *E* = −0.25 V and gives rise to two oxidative doping-induced polaronic bands, which, at *E* = +0.4 V, are located at 1385 and 797 nm. At this potential, the band at 638 nm, characteristic of the neutral state, is bleached.

**Figure 7:**
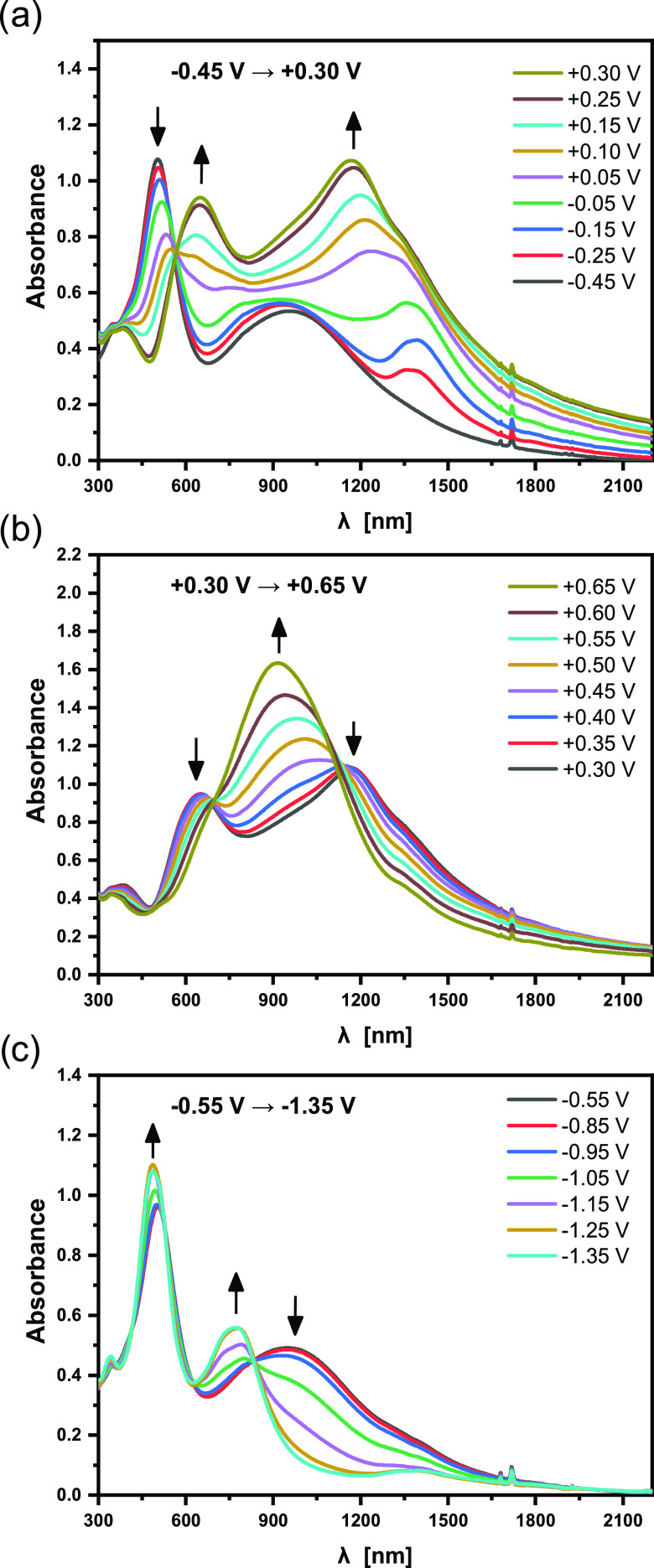
UV–vis–NIR spectra of a thin layer of **poly(DTP-NDI)** deposited on ITO, recorded for increasing electrode potential from −0.45 to +0.30 V (a) and from + 0.30 to +0.65 V (b) and decreasing from −0.55 to −1.35 V (c); electrolyte, 0.1 M Bu_4_PF_6_/CH_3_CN; potentials *vs* Fc/Fc^+^.

**Scheme 2:**
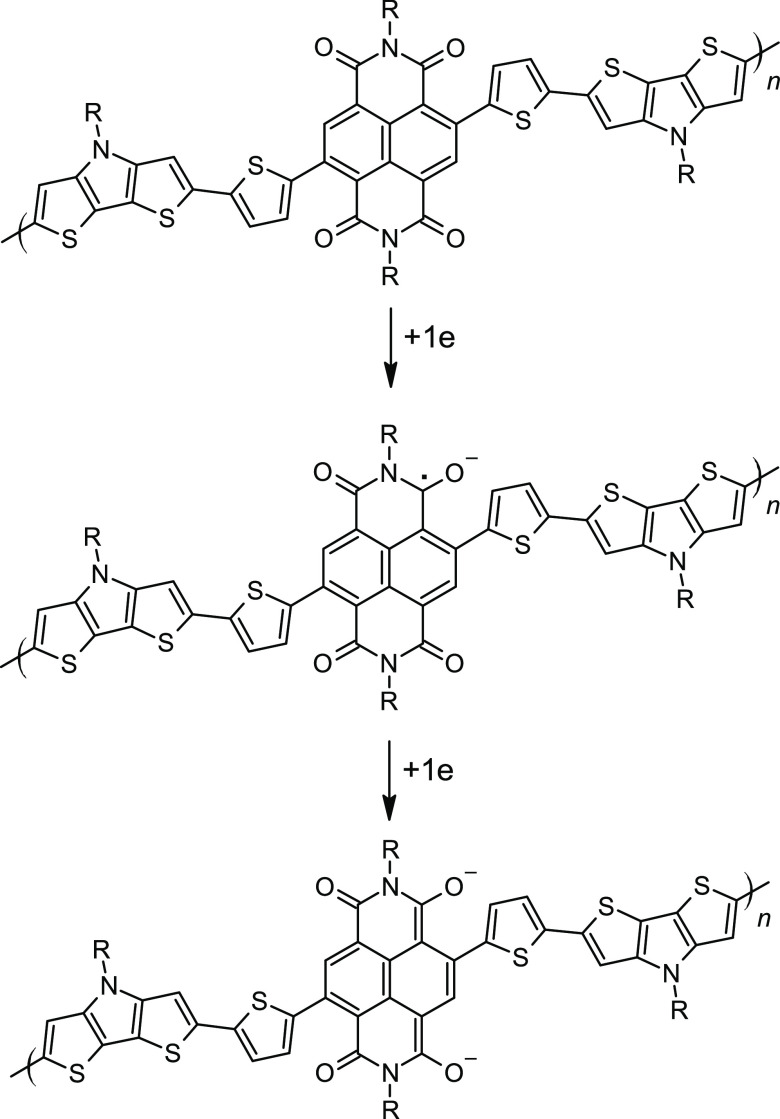
Formation of Radical Anion and Dianion Forms of **Poly(DTP-NDI)**

The spectroelectrochemistry of **poly(DTP-NDI)** deserves special interest since this polymer is characterized by four reversible or quasireversible redox couples covering a rather broad potential range from −1.4 to +0.60 V *vs* Fc/Fc^+^. Consequently, different oxidation states associated with these couples are characterized by distinctly different spectra. The prepared polymer shows features indicating its slight oxidation; therefore, registration of the spectrum of its neutral form requires polarization of the ITO electrode to *E* = −0.45 V. In Figure 7a, UV–vis–NIR spectral changes of **poly(DTP-NDI)** induced by the first oxidation process are compared. As in the case of **poly(DTP-TD**), the first oxidation process gives rise to spectral changes characteristic of conducting polymer oxidative doping. Thus, the lower energy (higher wavelength) oxidative doping-induced band appears at the onset potential of the first oxidation peak and undergoes a hypsochromic shift with increasing electrode potential, being located at 1168 nm at *E* = +0.3 V, *i.e.*, at the onset potential of the second oxidation peak. At lower potentials, the less-intensive band is somehow obscured because it is superimposed on the band ascribed to the neutral form of the polymer, but in the spectrum registered at *E* = +0.3 V, it is present as a clear peak with a maximum at 651 nm. In the potential range of the second oxidation peak (from *E* = +0.3 to +0.65 V), further spectral changes occur, leading to the merging of the two oxidative-doping-induced peaks into one broad band with a maximum at 915 nm at *E* = +0.65 V; all bands ascribed to the neutral form of the polymer were completely bleached (see Figure 7b). The spectroelectrochemical response of **poly(DTP-NDI)** in the reduction mode is distinctly different (see Figure 7c). As already stated, the neutral polymer is characterized by two bands in the UV–vis–NIR spectrum with maxima at 501 and 954 nm. Reduction-induced spectral changes appear at *E* = −0.95 V. At this potential, a relatively narrow peak appears and grows in intensity at the expense of the lower energy band (ascribed to the polymer neutral form). The higher energy band characteristic of the neutral polymer remains essentially intact. Its apparent increase in intensity is caused by its partial overlap with the reduction-induced band. At *E* = −1.35 V, the peak at 954 nm is totally bleached and the new band characteristic of the anionic form of the polymer is present as a distinct peak with a maximum 770 nm. It should be noted that these spectral features are different from those expected for a conducting polymer. Thus, it is instructive to discuss the differences between the charge configurations generated in **poly(DTP-NDI)** upon its oxidation and reduction. In the oxidized polymer, radical cations (positive polarons) and dications (positive bipolarons) constitute a part of a conjugated pathway extending over the polymer chain. This was demonstrated in our previous EPR spectroelectrochemical investigations of a polymer, which was very similar to **poly(DTP-NDI)**, namely, alternating copolymer consisting of the same acceptor (naphthalene diimide) and the same donor (dithienopyrrole), connected directly to the acceptor, *i.e.*, without the presence of a linker as in the case of the polymer studied in this research.^73^ Therefore, bands ascribed to polarons and/or bipolarons appear upon the oxidation. On the contrary, negative charges introduced to the neutral polymer chain upon its reduction lead to the formation of strongly localized radical anions and/or dianions, as shown in Scheme 2. For these reasons, in the oxidation mode, **poly(DTP-NDI)** behaves like a conducting polymer, whereas in the reduction mode, it behaves as a redox polymer.

The studied polymers seem to be promising candidates for electrochromic applications. Among them, **poly(DTP-NDI)** deserves special interest due to the high stability of its multiple redox states. For this reason, we have undertaken a detailed investigation of its electrochromism.

### 3.5 Electrochromism in Visible and NIR Spectral Ranges

Studies described in Section 3.4 clearly show that the spectroelectrochemical responses of the three polymers studied involve an extremely broad spectral range from UV–vis to NIR. These are interesting features since these optical features can find possible applications in devices exhibiting both UV–vis^34^ and NIR electrochromisms,^35^ provided that they exhibit good stability, leading to reproducible changes in color coordinates upon switching.

**Figure 8:**
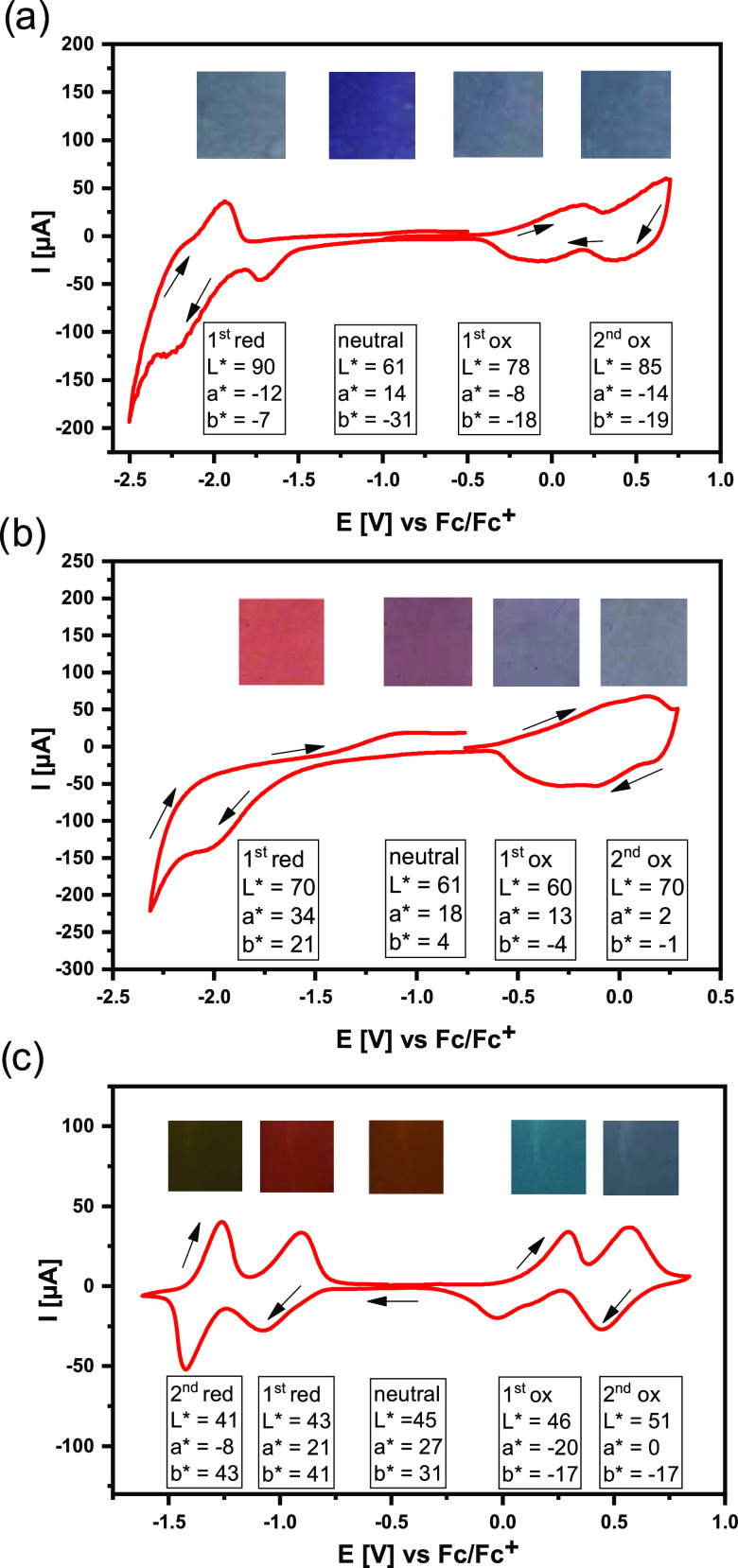
Cyclic voltammograms recorded for films of **poly(DTP-TD)** (a), **poly(DTP-TZ)** (b), and **poly(DTP-NDI)** (c) deposited on ITO electrodes, shown together with photos (clippings) illustrating their color changes and their color coordinates determined at different oxidation states; electrolyte, 0.1 M Bu_4_NPF_6_/ CH_3_CN; scan rate, 10 mV/s.

Cyclic voltammograms presented in Figure 8 clearly indicate that four different redox states can be attributed to **poly(DTP-DT)** and **poly(DTP-TZ)**, whereas in the case of **poly(DTP-NDI)**, five redox states can be distinguished. Additionally, CIE color coordinates corresponding to each particular redox state are presented together with photographs exhibiting visual color changes.

**Figure 9:**
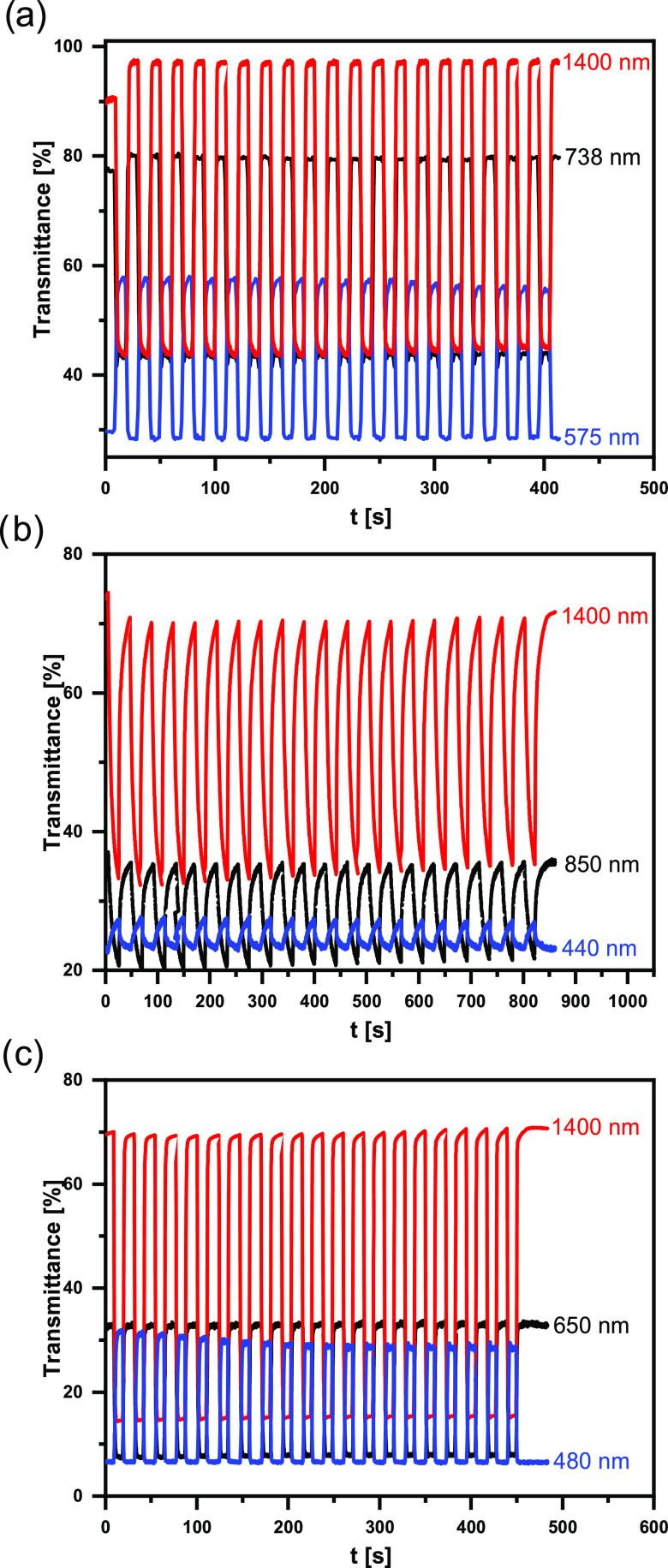
Transmittance changes recorded during electrochromic switching of polymer films: **poly(DTP-TD)** −0.7 V → 0.3 V (a), **poly(DTP-TZ)** −1.1 V → 0.1 V (b), and **poly(DTP-NDI)** −0.75 V → 0.35 V (c); electrolyte, 0.1 M Bu_4_NPF_6_/CH_3_CN; potentials *vs* Fc/Fc^+^.

The reversibility of transmittance changes upon potential switching is a crucial parameter to be determined for new electrochromic materials. In Figure 9, changes in the polymer film transmittance recorded for three different wavelengths of the radiation are presented. The three studied polymers were switched between the neutral and the first oxidized states (for identification of these states, see Figure 8). Since their oxidation from the neutral state to the first oxidized state involves profound changes in the NIR part of the spectrum, 1400 nm wavelength was selected for monitoring the oxidation-induced transmittance decrease. The other two wavelengths were selected individually for each polymer with the goal of assuring maximum transmittance changes upon switching.

The measured high reversibility of the transmittance clearly indicates the chemical stability of both neutral and first oxidized states. In general, dithienopyrrole donors in combination with appropriate acceptors yield promising electrochromic polymers. Moreover, the color of the studied polymers can be significantly modulated by the acceptor unit selection, as demonstrated not only in this research but also in previous reports.^74,75^ Thus, the studied polymers are promising visible and NIR electrochromes. Below, detailed descriptions of the electrochromic properties of the polymers tested are presented.

#### 3.5.1 Poly(DTP-TD)

**Poly(DTP-TD)** is characterized by its colored neutral state and more transparent oxidized and reduced states. This is clearly manifested by the evolution of their CIE color coordinates L*, a*, and b* (see Figure 8a). An increase of the lightness (L*), observed for the reduced and oxidized states, is associated with redox reaction-induced profound changes in the visible range of the spectrum. This involves bleaching of the principal absorption band characteristic of the neutral state, which covers a large part of the visible spectrum (see Figure 5). Extension of the number of switching cycles from 20 to 1000 cycles resulted in only negligible changes in the transmittance, proving the excellent electrochemical stability of this polymer (see Figure S7). Thus, thiadiazole, if combined with dithienopyrrole in a polymer of the -(D-π-A-π-D)- type, turns out to be an interesting electrochromic material of excellent switching stability, superior to that reported for electrochromes containing oxadiazole acceptors.^76,77^

#### 3.5.2 Poly(DTP-TZ)

Electrochemical oxidation of **poly(DTP-TZ)**, as probed by cyclic voltammetry, gives rise to two strongly overlapping redox couples (see Figure 8b). They correspond to two oxidation states of different electrochromic parameters. Upon oxidation from the neutral to the first oxidized state, no significant changes in transmittance are observed. Measurable modifications of the CIE color parameters can be noticed, resulting mainly from partial bleaching of the principal absorption band located in the visible part of the spectrum and characteristic of the neutral state of the polymer (see Figure 6). Further oxidation to the second oxidized state leads to an increase in the polymer film transmittance and further modification of the CIE coordinates. The reduction of neutral **poly(DTP-TZ)** to its anionic state also results in marked color changes (Figure 8b) and in an increase of transmittance. Switching between the neutral and reduced states, however, shows poor reversibility. This is mainly associated with low stability of the anionic form of **poly(DTP-TZ)** and charge trapping upon its dedoping.^78^

#### 3.5.3 Poly(DTP-NDI)

Five different oxidation states can be distinguished in the case of **poly(DTP-NDI)**: neutral, two oxidized, and two reduced states (see their cyclic voltammograms in Figures 4 and 8). Switching between these states results in small modifications of L* and more significant changes in a* and b* parameters (Figure 8**c**). Upon oxidation of neutral **poly(DTP-NDI)** to its first oxidized form, a* and b* parameters change signs from positive to negative, representing red-green and yellow-blue opposite color shifts. Further oxidation to the second oxidized state results in an increase of a* from −17 to 0 with b* remaining unchanged. Changes in a* and b* parameters induced by the reduction of the neutral polymer to its first reduced state are surprisingly small (Figure 8c). This is caused by the fact that the first reduction process rather induces a minor modification of the visible range of the spectrum, the principal spectral changes involving the NIR spectral region. This is clearly seen in Figure 7c presenting the spectroelectrochemical data collected in the reduction mode. Switching to the second reduced state results in a noticeable change of a* accompanied by an insignificant increase of b*. It should be noted that the most profound spectral changes occur in the NIR part of the spectrum, but they do not contribute to visible color changes. Switching involving the second reduced state is of limited stability, consistent with previous reports on alternating copolymers of naphthalene diimide and carbazole or fluorene.^79^

The electrochromic behavior of **poly(DTP-NDI)** described in this research is in line with previous reports of electrochromism of -(D-A-D)- polymers consisting of the naphthalene diimide acceptor and carbazole,^38^ triphenylamine,^80^ or phenothiazine^81^ donors. All of these polymers exhibit a rich color palette originating from the fact that due to their ambipolar nature, their electrochromism can exploit different oxidation as well as reduction states. Significant differences in the charge storage configuration in the reduced and oxidized states of these polymers should be pointed out. Surplus electrons introduced during the reduction are localized on naphthalene diimide units, as shown in Scheme 2, absorbing mainly visible light.^38,79,82^ Radical cations and dications formed upon their oxidation are, in turn, delocalized along the conjugated polymer backbone, as in conducting polymers, contributing mainly to the changes in the NIR region of the spectrum.^6^

## 4 Conclusions

To summarize, we carried out joint experimental and theoretical studies on five D-π-A-π-D compounds consisting of dithieno[3,2-*b*:2′,3′-*d*]pyrrole (**DTP**) donors connected *via* 2,5-thienylene linkers to acceptors of increasing electron-withdrawing ability, namely, 1,3,4-thiadiazole (**TD**) benzo[*c*][1,2,5]thiadiazole (**BTD**), 2,5-dihydropyrrolo[3,4-*c*]pyrrole-1,4-dione (**DPP**), 1,2,4,5-tetrazine (**TZ**), and benzo[*lmn*][3,8]phenanthroline-1,3,6,8(2*H*,7*H*)-tetraone (**NDI**). The acceptor strength had a very pronounced effect on optical and redox properties of the studied compounds bathochromically shifting their absorption and emission bands and increasing their electron affinities (|EA|s). Ionization potentials were affected to a much lesser extent. Three of the studied compounds (**DTP-TD, DTP-BTD**, and **DTP-DPP**) turned out to be interesting luminophores exhibiting emission spectra whose energy and quantum yields (PLQYs) were strongly dependent on the solvent polarity. The above outlined phenomena were strongly supported by DFT calculations, which correctly predicted the dependence of the absorption and emission bands energies and PLQYs values on solvent polarity. The same applies to adiabatic IP and EA values, elegantly predicted by DFT calculations and remaining in very close agreement with the experiment.

Four compounds (**DTP-TD**, **DTP-BTD**, **DTP-TZ**, and **DTP-NDI**) readily electropolymerized, yielding polymers of very narrow electrochemical band gaps approaching values of *ca.* 1 eV in the case of **poly(DTP-TZ)** and **poly(DTP-NDI)**. **Poly(DTP-TD)**, **poly(DTP-TZ)**, and **poly(DTP-NDI)** turned out to be interesting electrochromic materials showing four or five redox states differing in color coordinates and lightness.

## Supporting Information Available

Synthesis and characterization of compounds; absorption and emission data; differential pulse voltammograms; isosurface of the spin densities; calculated molecular structure details; electropolymerization curves; and transmittance changes induced by electrochromic switching (PDF)
