# Supplementary material for: D-A-D Compounds Combining Dithienopyrrole Donors and Acceptors of Increasing Electron-Withdrawing Capability: Synthesis, Spectroscopy, Electropolymerization, and Electrochromism
Source: J Phys Chem B. 2022 May 26;126(22):4089–105. doi: 10.1021/acs.jpcb.2c01772 (PMC9189846; doi:10.1021/acs.jpcb.2c01772)
Supplement: Supplementary file 1 — jp2c01772_si_001.pdf [file jp2c01772_si_001.pdf]

Supporting Information

D-A-D Compounds Combining Dithienopyrrole Donors and Acceptors of Increasing Electron Withdrawing Capability: Synthesis, Spectroscopy, Electropolymerization and Electrochromism

Renata Rybakiewicz-Sekita[1,2]*, Petr Toman[3], Roman Ganczarczyk[1], Jakub Drapala[1], Przemyslaw Ledwon[4], Marzena Banasiewicz[5], Lukasz Skorka[1], Anna Matyjasiak[1], Malgorzata Zagorska[1]*, Adam Pron[1]*

[1]Faculty of Chemistry, Warsaw University of Technology, Noakowskiego 3, 00-664 Warsaw, Poland

[2]Faculty of Mathematics and Natural Sciences. School of Sciences, Institute of Chemical Sciences, Cardinal Stefan Wyszynski University in Warsaw, Woycickiego 1/3, 01-815 Warsaw, Poland

[3]Institute of Macromolecular Chemistry, Academy of Sciences of the Czech Republic, Heyrovsky Sq. 2, 162 06 Prague 6, Czech Republic

[4]Faculty of Chemistry, Silesian University of Technology, Strzody 9, 44-100 Gliwice, Poland

[5]Institute of Physics, Polish Academy of Sciences, Al. Lotnikow 32/44, 02-668 Warsaw, Poland

1. Synthesis and Characterization S2
2. Spectroscopic Studies S9
3. Electrochemical Studies S11
4. Theoretical Studies S12
5. Electropolymerization S17
6. Electrochromism S19
7. References S19

1. Synthesis and Characterization

Materials

Substrates used for the preparation of the final compounds, namely 4-(2-ethylhexyl)-2-(tributylstannyl)-4*H*-dithieno[3,2-*b*:2',3'-*d*]pyrrole (DTP-$SnBu_3$) and dibromoderivatives of the corresponding acceptors *i.e.* 2,5-bis(5-bromothiophen-2-yl)-1,3,4-thiadiazole (**Br-DTTD-Br**), 4,7-bis(5-bromothiophen-2-yl)benzo[*c*][1,2,5]thiadiazole (**Br-DTBTD-Br**), 3,6-bis(5-bromothiophen-2-yl)-1,2,4,5-tetrazine (**Br-DTTZ-Br**) and 3,6-bis(5-bromothiophen-2-yl)-2,5-bis(2-ethylhexyl)-2,5-dihydropyrrolo[3,4-*c*]pyrrole-1,4-dione (**Br-DTDPP-Br**) were prepared by procedures described in the literature [1],[2],[3],[4] or by their minor modifications. Chemicals and dry solvents for all syntheses were purchased from Sigma-Aldrich, whereas solvents for extraction and column chromatography were delivered by CHEMPUR. Column chromatography was performed using Merck silica gel 60 (0.040 – 0.063 mm). Zeonex 480 was generously donated by Zeon Europe GmbH. All reactions were carried out under dry argon.

Characterization techniques

^{1}H and ^{13}C NMR spectra were recorded on a Varian Mercury (500 and 125 MHz) spectrometer. Mass spectra were measured by EI method on an AMD 604 mass spectrometer. All final products were subject to C, H, N and S elemental analysis. IR spectra were recorded on a Nicolet iS5 ThermoScientific spectrometer (ATR).

Thin films preparation

Zeonex pellets (296 mg) were stirred in toluene (2.960 ml) for 2h in a sealed vial at elevated temperature (50°C). **DTP-TD** (3.11 mg) was dissolved in toluene (3.110 ml). The resulting solutions were mixed in a volume ratio providing appropriate concentration of the compound in Zeonex dispersion. The obtained mixtures were deposited on quartz substrates and heated to 70°C to evaporate the solvent. The resulting thin films were additionally dried in vacuo.

2,5-bis(5-(4-(2-ethylhexyl)-4H-dithieno[3,2-b:2',3'-d]pyrrol-2-yl)thiophen-2-yl)-1,3,4-thiadiazole (DTP-TD)

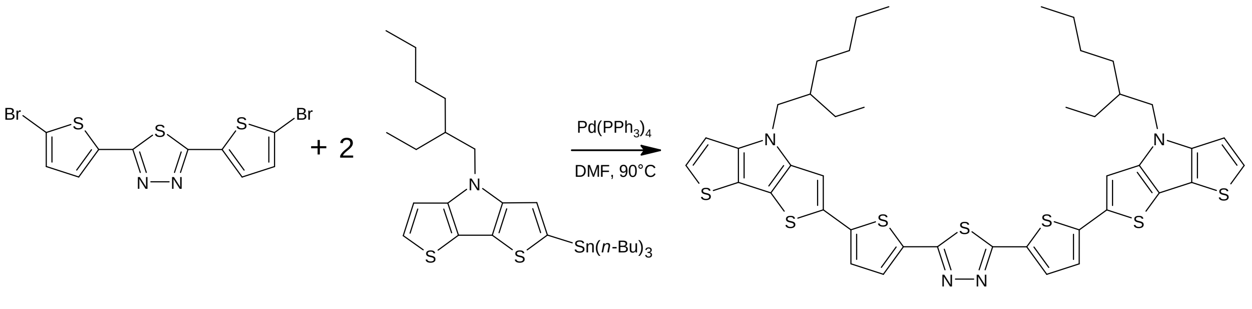

$Pd(PPh_3)_4$ (0.53 g, 0.0458 mmol) was added to a suspension of 2,5-bis(5-bromo-2-thienyl)-1,3,4-thiadiazole (0.187 g, 0.458 mmol) in dry DMF (25 ml). The mixture was stirred to afford clear yellow solution. Then, a solution of DTP-Sn(*n*-Bu)$_3$ (0.568 g, 0.978 mmol) in DMF (5 ml) was added dropwise over the course of 0.5 h. The mixture was heated to 90 °C and stirred for 2 h. The resulting reddish-brown solution was allowed to cool to ambient temperature and then washed with brine. The aqueous phase was extracted with dichloromethane (DCM) (3 x 50 ml). Combined organic phases were dried over anhydrous $MgSO_4$, concentrated and purified by silica column chromatography using hexane/DCM (1:1) eluent to obtain reddish-brown solid (0.271 g, 71%).

Melting point: 184 – 186 °C. **^{1}H NMR** (500 MHz, $CDCl_3$), δ: 7.30 (d. J = 3.9 Hz. 2H). 7.16 (d. J = 5.3 Hz. 2H). 7.11 (s. 2H). 7.07 (d. J = 3.9 Hz. 2H). 6.93 (d. J = 5.3 Hz. 2H). 4.05 – 3.94 (m. 4H). 1.97 – 1.88 (m. 2H). 1.39 – 1.26 (m. 16H). 0.91 (t. J = 7.4 Hz. 6H). 0.89 (t. J = 7.0 Hz. 6H). **^{13}C NMR** (126 MHz, $CDCl_3$) δ 160.52. 146.06. 145.15. 143.07. 133.12. 130.46. 129.47. 124.31. 123.12. 115.09. 114.87. 111.21. 108.83. 51.43. 40.58. 30.75. 28.77. 24.18. 23.15. 14.20. 10.85. **IR** (wavenumber, cm^{-1}) 3068, 2953, 2923, 2855, 1524, 1497, 1435, 1409, 1377, 1253, 1225, 1203, 1092, 1061, 921, 890, 833, 789, 703, 639, 600, 523, 477, 450. **Elemental analysis** calcd C 60.83, H 5.35, N 6.76, S 27.07; found C 61.09, H 5.38, N 6.94, S 26.57. **HRMS (EI+)** calcd for $C_{42}H_{44}N_4S_7$: 828.1611; found: 828.1649. **UV-vis,** ε [M^{-1}·cm^{-1}] = 85271 (478 nm).

4,7-bis(5-(4-(2-ethylhexyl)-4H-dithieno[3,2-b:2',3'-d]pyrrol-2-yl)thiophen-2-yl)benzo[c][1,2,5]thiadiazole (DTP-BTD)

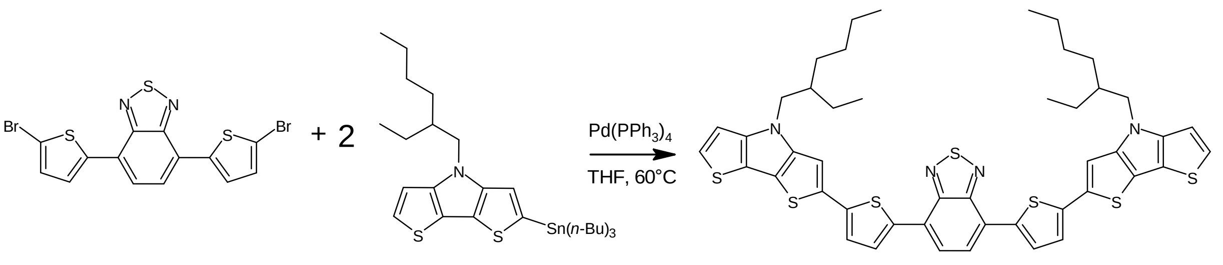

$Pd(PPh_3)_4$ (0.080 g, 0.069 mmol) was added to a suspension of 4,7-bis(5-bromothiophen-2-yl)-2,1,3-benzothiadiazole (0.201 g, 0.439 mmol) in dry tetrahydrofuran (THF) (25 ml). The mixture was stirred at room temperature to afford clear red solution. A solution of DTP-Sn(*n*-Bu)$_3$ (0.607 g, 1.045 mmol) in THF (5 ml) was added dropwise over the course of 0.5 h. The mixture was then heated to 60°C and then stirred for 24 h. The resulting solution was allowed

to cool to ambient temperature and then washed with brine. The aqueous phase was extracted with dichloromethane DCM (3 × 50 ml). Combined organic phases were dried over anhydrous $MgSO_4$, concentrated and purified by silica column chromatography using hexane/DCM (3:1) eluent to obtain dark violet solid (0.090 g, 23 %).

Melting point >250°C. **^{1}H NMR (500 MHz, $CDCl_3$)** δ 7.98 (d. *J* = 3.9 Hz. 2H). 7.76 (s. 2H). 7.22 (d. *J* = 3.9 Hz. 2H). 7.15 (d. *J* = 5.4 Hz. 2H). 7.13 (s. 2H). 6.94 (d. *J* = 5.3 Hz. 2H). 4.05 – 3.95 (m. 4H). 2.00 – 1.91 (m. 2H). 1.39 – 1.27 (m. *J* = 13.3. 9.3. 7.0 Hz. 16H). 0.92 (t. *J* = 7.4 Hz. 6H). 0.90 (t. *J* = 7.1 Hz. 6H). **^{13}C NMR (126 MHz, $CDCl_3$)** δ 152.53. 145.63. 145.32. 140.58. 137.43. 134.36. 128.30. 125.36. 124.97. 123.71. 123.68. 114.97. 114.34. 111.20. 107.93. 51.47. 40.54. 30.76. 28.78. 24.20. 23.18. 14.23. 10.87. **IR** (wavenumber, cm^{-1}) 3103, 3081, 2954, 2922, 2854, 1520, 1497, 1464, 1430, 1409, 1361, 1292, 1226, 1204, 1092, 1060, 907, 873, 836, 787, 702, 641, 609, 590, 543, 505, 470, 450. **Elemental analysis**: calcd C 62.83, H 5.27, N 6.37, S 25.53; found C 62.87, H 5.29, N 6.38, S 24.73. **HRMS (EI):** calcd for $C_{46}H_{46}N_4S_7$: 878.1734; found: 878.1755. **UV-vis,** ε [$M^{-1}·cm^{-1}$] = 41974 (564 nm), 43670 (400 nm).

3,6-bis(5-(4-(2-ethylhexyl)-4H-dithieno[3,2-b:2',3'-d]pyrrol-2-yl)thiophen-2-yl)-1,2,4,5-tetrazine (DTP-TZ)

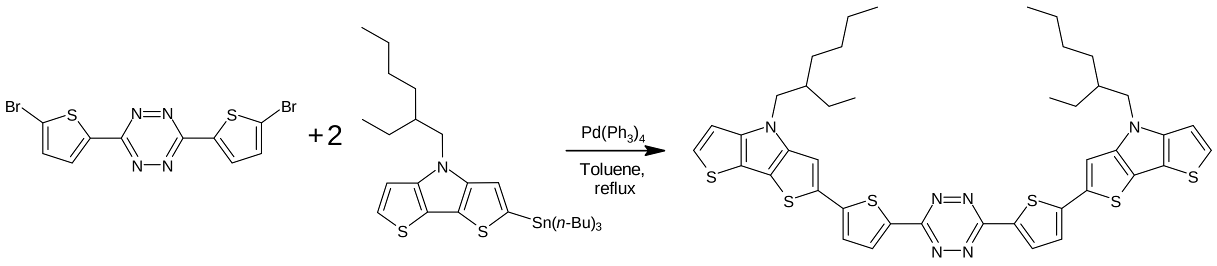

$Pd(PPh_3)_4$ (0.114 g, 0.099 mmol) was added to a suspension of 3,6-bis(5-bromothiophen-2-yl)-1,2,4,5-tetrazine (0.400 g, 0.990 mmol) in dry toluene (30 ml). The mixture was stirred to afford clear orange solution. Then, a solution of DTP-Sn(*n*-Bu)$_3$ (1.321 g, 2.277 mmol) in toluene (10 ml) was added dropwise over the course of 0.5 h. The mixture was heated to reflux and stirred for 20 h. The resulting orange solution was allowed to cool to ambient temperature and then concentrated. Crude product was purified by silica column chromatography using hexane/DCM (2:1) eluent followed by crystallization from chloroform/hexane mixture yielding 0.500 g of dark green crystals (0.500 g, 61%).

Melting point: 183 – 185°C. **^{1}H NMR (500 MHz, $CDCl_3$)** δ 8.09 (d, J = 3.9 Hz, 2H), 7.25 (s, 2H), 7.20 (s, 2H), 7.17 (d, J = 5.2 Hz, 2H), 6.95 (d, J = 5.3 Hz, 2H), 4.10 – 3.99 (m, 4H), 1.99 – 1.92 (m, 2H), 1.41 – 1.25 (m, 16H), 0.93 (t, J = 7.5 Hz, 6H), 0.90 (t, J = 7.0 Hz, 6H). **^{13}C NMR (126 MHz, $CDCl_3$)** δ 160.81, 146.31, 146.12, 145.25, 133.34, 133.14, 131.80, 124.68, 124.12, 115.64, 114.94, 111.19, 109.16, 51.52, 40.61, 30.78, 28.79, 24.21, 23.16, 14.21, 10.86. **IR** (wavenumber, cm^{-1}) 3098, 3076, 2955, 2923, 2848, 1274, 1545, 1527, 1495, 1429, 1392, 1274, 1224, 1139, 1092, 1075, 1057, 998, 918, 841, 817, 793, 762, 704, 643, 604, 524, 487, 450. **Elemental analysis** calcd C 61.13, H 5.37, N 10.18, S 23.31; found C 60.38; H 5.46; N 10.05; S 22.81. **HRMS (TOF MS ES+):** calcd for $C_{42}H_{44}N_6S_6$ 824.1952; found 824.1919. **UV-vis,** ε [M^{-1}·cm^{-1}] = 82355 (519 nm).

2,5-bis(2-ethylhexyl)-3,6-bis(5-(4-(2-ethylhexyl)-4H-dithieno[3,2-b:2',3'-d]pyrrol-2-yl) thiophen-2-yl)-2,5-dihydropyrrolo[3,4-c]pyrrole-1,4-dione (DTP-DPP)

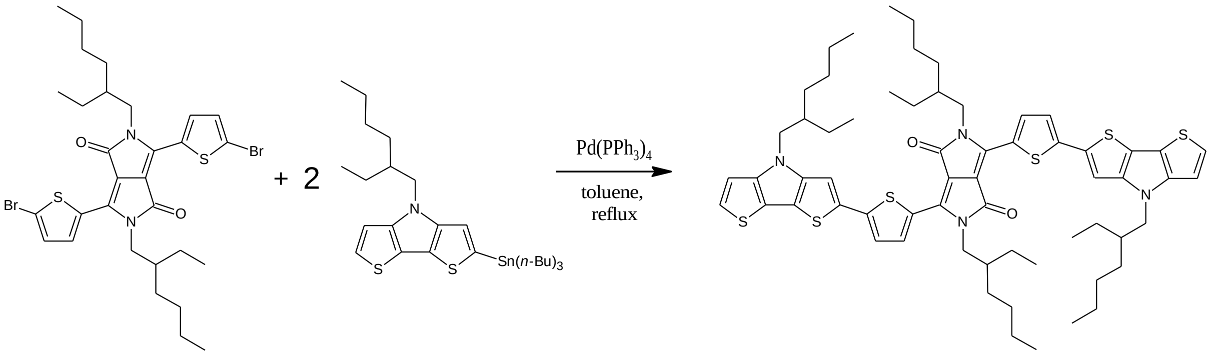

$Pd(PPh_3)_4$ (0.058 g, 0.050 mmol) and a solution of DTP-Sn(*n*-Bu)$_3$ (0.269 g, 0.463 mmol) in dry toluene (5 ml) were added to a solution of 3,6-bis(5-bromo-2-thienyl)-2,5-bis(2-ethylhexyl)-2,5-dihydropyrrolo[3,4-*c*]pyrrole-1,4-dione (0.140 g, 0.205 mmol) in dry toluene (40 ml). The resulting mixture was then heated to reflux and stirred for 5 h. After cooling to ambient temperature, the dark blue solution was concentrated. Crude product was purified in two steps using silica chromatographic columns. Hexane/DCM (1:3) and hexane/ DCM (1:1) were used as eluents, in the first and the second step, respectively. Dark blue powder was obtained (0.073 g, 32%).

Melting point: > 250°C. **^{1}H NMR (500 MHz, $CDCl_3$)** δ 8.93 (d, J = 3.9 Hz, 2H), 7.28 (d, J = 4.1 Hz, 2H), 7.18 (d, J = 5.2 Hz, 2H), 7.14 (s, 2H), 6.94 (d, J = 5.2 Hz, 2H), 4.12 – 3.94 (m, 8H), 1.99 – 1.89 (m, 4H), 1.44 – 1.24 (m, 32H), 0.97 – 0.86 (m, 24H). **^{13}C NMR (126 MHz,**

$CDCl_3$) δ 161.75, 146.33, 145.41, 144.56, 139.05, 136.78, 133.14, 127.53, 124.69, 123.79, 115.65, 115.00, 111.21, 108.76, 108.31, 51.47, 46.12, 40.54, 39.43, 30.75, 30.56, 28.76, 28.72, 24.17, 23.89, 23.30, 23.14, 14.30, 14.19, 10.84, 10.80. **IR** (wavenumber, cm^{-1}) 3077, 3069, 3052, 2956, 2925, 2872, 2854, 1643, 1543, 1519, 1442, 1421, 1404, 1394, 1364, 1309, 1248, 1221, 1160, 1069, 1017, 917, 844, 789, 730, 707, 646, 591, 470, 451. **Elemental analysis**, calcd. C 67.51, H 7.08, N 5.08, S 17.42; found C 68.24; H 7.44; N 4.81; S 16.66. **HRMS (EI+):** calcd for $C_{62}H_{78}N_4O_2S_6$ 1102.4449, found 1102.4747. **UV-vis,** ε [$M^{-1}\cdot cm^{-1}$] = 89912 (672 nm).

2,7-bis(2-ethylhexyl)-4,9-bis(5-(4-(2-ethylhexyl)-4H-dithieno[3,2-b:2',3'-d]pyrrol-2-yl)thiophen-2-yl)benzo[lmn][3,8]phenanthroline-1,3,6,8(2H,7H)-tetraone (DTP-NDI)

a) *2,6-bis(thiophen-2-yl)-N,N'-bis(2-ethylhexyl)-1,4,5,8-naphthalene-tetracarboxylic acid dimide* ***(DTNDI)***

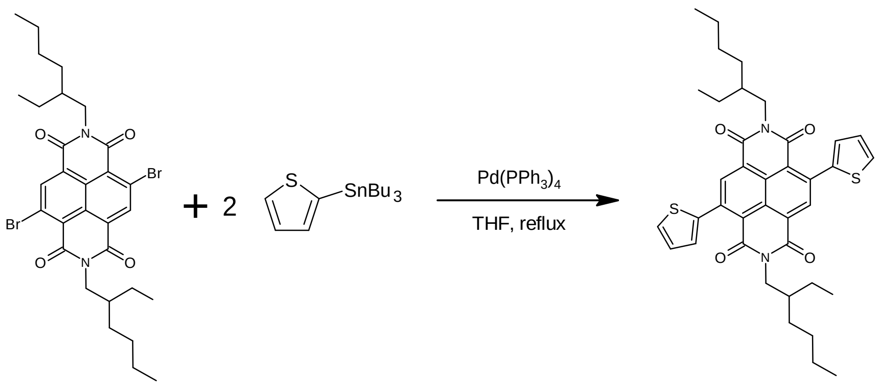

$Pd(PPh_3)_4$ (0.89 g, 0.077 mmol) and 2-tributylstannylthiophene (0.690 g, 1.849 mmol) were added to a suspension of *N,N'*-bis(2-ethylhexyl)-2,6-dibromo-1,4,5,8-naphthalenetetracarboxylic acid diimide (0.500 g, 0.771 mmol) in dry tetrahydrofuran (60 ml). The mixture was heated to reflux and stirred for 24 h. The resulting brown solution was allowed to cool to ambient temperature and then organic solvent was evaporated. Crude product was purified by crystallization from methanol, yielding red powder (0.480 g, 95%).

^{1}H NMR (500 MHz, $CDCl_3$) δ 8.76 (s, 2H), 7.57 (dd, *J* = 5.1, 1.2 Hz, 2H), 7.30 (dd, *J* = 3.6, 1.2 Hz, 2H), 7.20 (dd, *J* = 5.1, 3.6 Hz, 2H), 4.13 – 4.02 (m, 4H), 1.95 – 1.86 (m, 2H), 1.39 – 1.23 (m, 16H), 0.90 (t, *J* = 7.4 Hz, 6H), 0.87 (t, *J* = 7.0 Hz, 6H). **^{13}C NMR (126 MHz, $CDCl_3$)** δ 162.75, 162.51, 140.96, 140.37, 136.81, 128.39, 128.22, 127.62, 127.58, 125.54, 123.53, 44.72, 37.86, 30.75, 28.70, 24.05, 23.23, 14.23, 10.76.

b) *4,9-bis(5-bromothiophen-2-yl)-2,7-bis(2-ethylhexyl)benzo[lmn][3,8]phenanthroline-1,3,6,8(2H,7H)-tetraone* ***(Br-DTNDI-Br)***

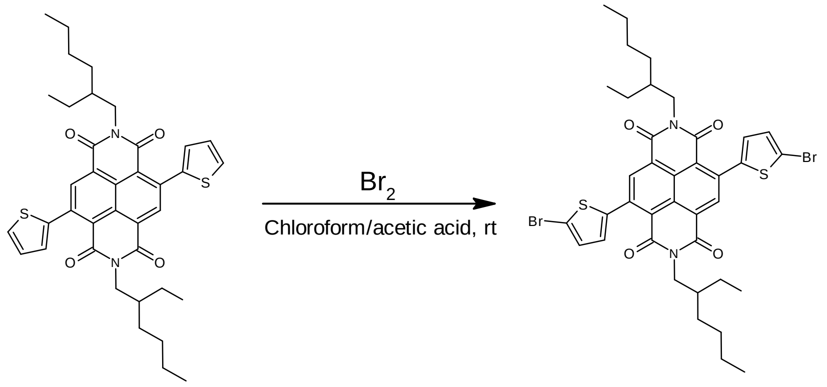

2,6-bis(thiophen-2-yl)-*N,N'*-bis(2-ethylhexyl)-1,4,5,8-naphthalene-tetracarboxylic acid diimide (0.450 g, 0.687 mmol) was dissolved in 150 ml mixture of chloroform and acetic acid 5:1 (*v/v*). Then, Br_2 (0.878 g, 5.496 mmol) was added in two portions. The mixture was stirred for 2h at room temperature. The crude product was washed with water, then with saturated solution of sodium thiosulfate and solution of sodium bicarbonate. The organic phase was concentrated and passed through silica pad using hexane/ DCM (1:2) eluent. Crude product was crystalized from chloroform/hexane mixture, yielding dark red powder (0.460 g, 82%).

^{1}H NMR (500 MHz, $CDCl_3$) δ 8.71 (s, 2H), 7.15 (d, *J* = 3.8 Hz, 2H), 7.08 (d, *J* = 3.8 Hz, 2H), 4.12 – 4.03 (m, 4H), 1.95 – 1.84 (m, 2H), 1.40 – 1.22 (m, 16H), 0.91 (t, *J* = 7.4 Hz, 6H), 0.87 (t, *J* = 7.0 Hz, 6H). **^{13}C NMR (126 MHz, $CDCl_3$)** δ 162.49, 162.43, 142.29, 139.21, 136.60, 130.35, 128.98, 127.68, 125.76, 123.29, 115.67, 44.80, 37.90, 30.74, 28.65, 24.06, 23.24, 14.24, 10.76.

c) *2,7-bis(2-ethylhexyl)-4,9-bis(5-(4-(2-ethylhexyl)-4H-dithieno[3,2-b:2',3'-d]pyrrol-2-yl)thiophen-2-yl)benzo[lmn][3,8]phenanthroline-1,3,6,8(2H,7H)-tetraone* ***(DTP-NDI)***

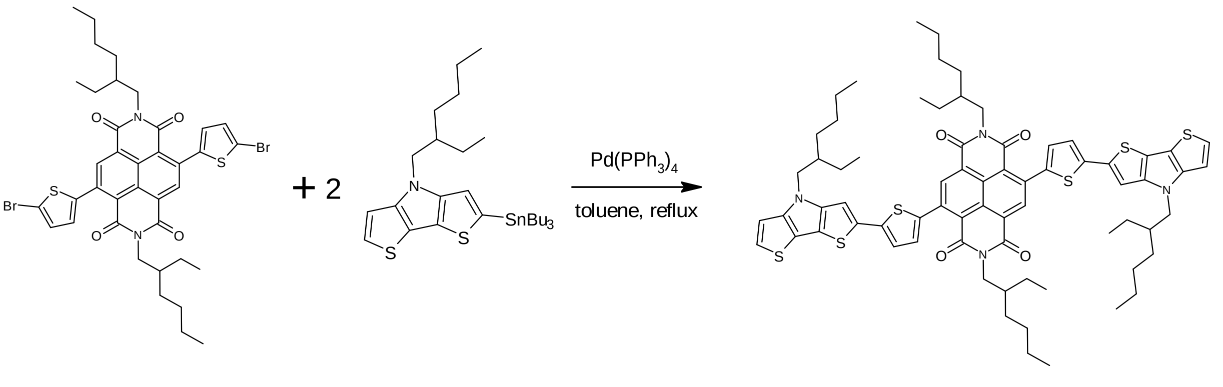

2,6-bis(5-bromothiophen-2-yl)-*N,N'*-bis(2-ethylhexyl)-1,4,5,8-naphthalene-tetracarboxylic acid dimide (0.300 g, 0.369 mmol) was dissolved in dry toluene (35 ml). Then $Pd(PPh_3)_4$ (0.042 g, 0.037 mmol) and DTP-Sn(*n*-Bu)$_3$ (0.471 g, 0.812 mmol) in toluene (10 ml) were added. The reaction mixture was heated to reflux and stirred for 20 h. The resulting solution was allowed to cool to ambient temperature and then washed with saturated brine. The aqueous phase was extracted with DCM (3 × 50 ml). Combined organic phases were dried over anhydrous $MgSO_4$, concentrated and purified by silica column chromatography using hexane/DCM (2:3) eluent to obtain green solid (0.268 g, 59 %).

Melting point: decomposes before melting. **^{1}H NMR (500 MHz, $CDCl_3$)** δ 8.79 (s, 2H), 7.35 (d, *J* = 3.8 Hz, 2H), 7.26 (d, *J* = 3.8 Hz, 2H), 7.17 (d, *J* = 5.3 Hz, 2H), 7.14 (s, 2H), 4.17 – 4.00 (m, 8H), 2.01 – 1.91 (m, 4H), 1.44 – 1.24 (m, 32H), 0.97 – 0.86 (m, 24H). **^{13}C NMR (126 MHz, $CDCl_3$)** δ 162.83, 162.76, 145.78, 145.23, 142.78, 139.32, 138.76, 136.64, 133.85, 130.70, 127.66, 125.38, 124.05, 123.32, 122.22, 114.96, 114.85, 111.17, 108.46, 51.53, 44.77, 40.58, 37.95, 30.83, 30.79, 28.80, 28.78, 24.19, 24.10, 23.27, 23.15, 14.28, 14.20, 10.84, 10.78. **IR** (wavenumber, cm^{-1}) 3101, 3082, 2955, 2924, 2856, 1703, 1660, 1570, 1525, 1489, 1425, 1379, 1309, 1262, 1198, 1155, 1093, 1062, 910, 800, 787, 711, 652, 567, 552, 498, 451. **Elemental analysis**: calcd. C 68.10, H 6.54, N 4.54, S 15.59; found: C 68.59, H 6.78, N 4.52, S 15.67. ε [$M^{-1}\cdot cm^{-1}$] = 21487 (661 nm), 79162 (389 nm). **MALDI-TOF-MS**: mass calcd for $C_{70}H_{80}N_4O_4S_6$ 1232.4504, found 1232.473.

2. Spectroscopic Studies

Table S1. Absorption and emission properties of **DTP-TD, DTP-BTD** and **DTP-DPP** in different solvents.

**Compound**	Solvent	$\lambda_{max}^{abs}/\lambda_{max}^{em}$ [nm]	ϕ [%]
**DTP-TD**	dichloromethane	478/585	55[a]
	toluene	477/543	64[a]
	acetonitrile	472/612	41[a]
	tetrahydrofuran	478/587	53[a]
	dimethyl sulfoxide	487/635	36[a]
	n-hexane	469/522	67[a]
**DTP-BTD**	dichloromethane	564/775	19[b]
	toluene	565/713	50[b]
	acetonitrile	552/820	4.8[b]
	tetrahydrofuran	569/756	31[b]
	dimethyl sulfoxide	576/860	2.6[b]
	n-hexane	563/670	37[b]
**DTP-DPP**	dichloromethane	672/722	19[b]
	toluene	674/709	22[b]
	tetrahydrofuran	673/716	17[b]

[a] Rh6G in EtOH as a standard, [b] Rh800 in EtOH as a standard

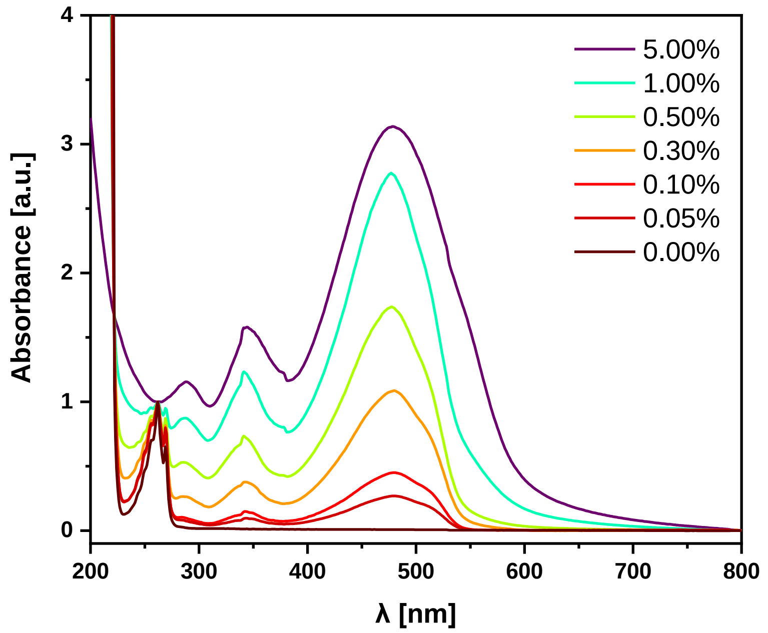

Figure S1. Absorption spectra of **DTP-TD** molecularly dispersed in Zeonex. The spectra are normalised at the wavelength of 262 nm *i.e.* the maximum of the matrix diagnostic absorption band.

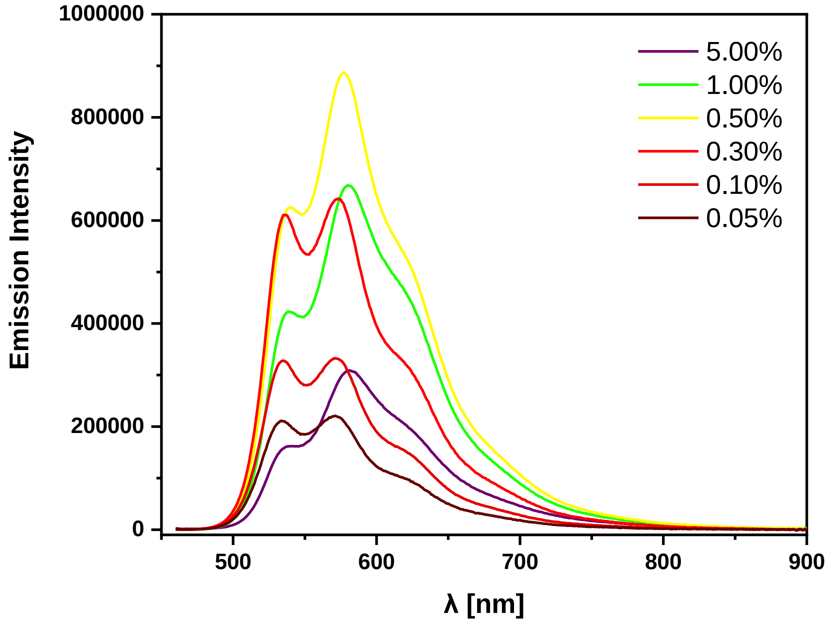

Figure S2. Emission spectra of **DTP-DT** molecularly dispersed in Zeonex. Excitation wavelength: 450 nm.

3. Electrochemical Studies

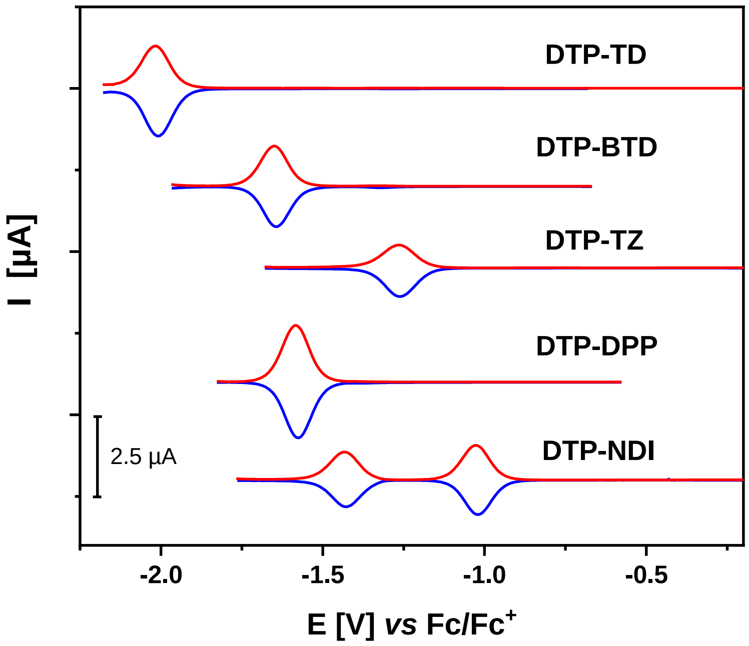

Figure S3. Differential pulse voltammograms of: **DTP-TD, DTP-BTD**, **DTP-TZ**, **DTP-DPP** and **DTP-NDI**. Concentration 10^{-3} M; electrolyte 0.1 M Bu_4NPF_6/CH_2Cl_2.

4. Theoretical Studies

cation-radical **anion-radical**

DTP-TD

DTP-BTD

DTP-TZ

DTP-DPP

DTP-NDI

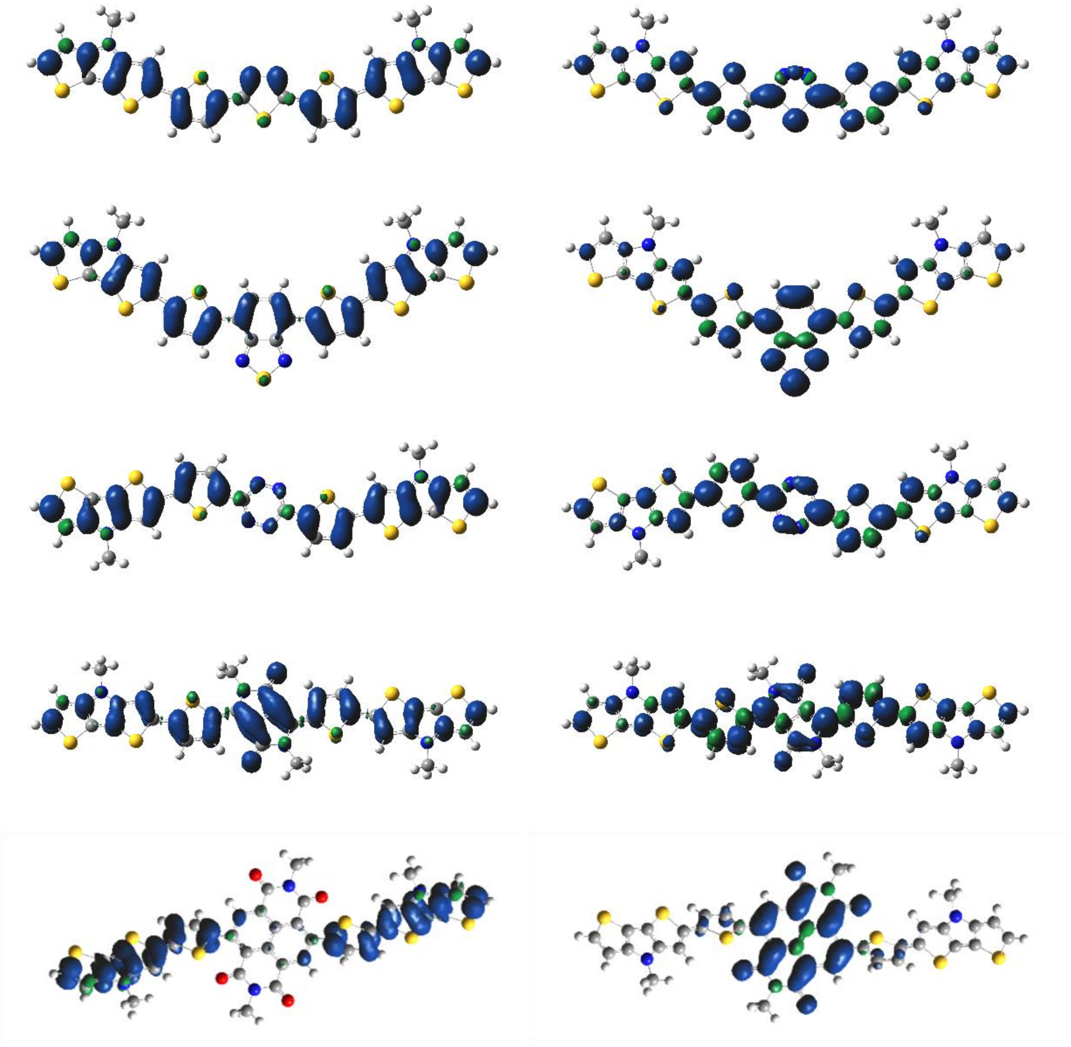

Figure S4. Isosurfaces of the spin densities of ion-radicals of the studied derivatives in **vacuum,** isovalue = 0.0004 a.u. Positive spin density values are displayed in blue and negative ones in green. Method: B3LYP-D3/6-311+G*.

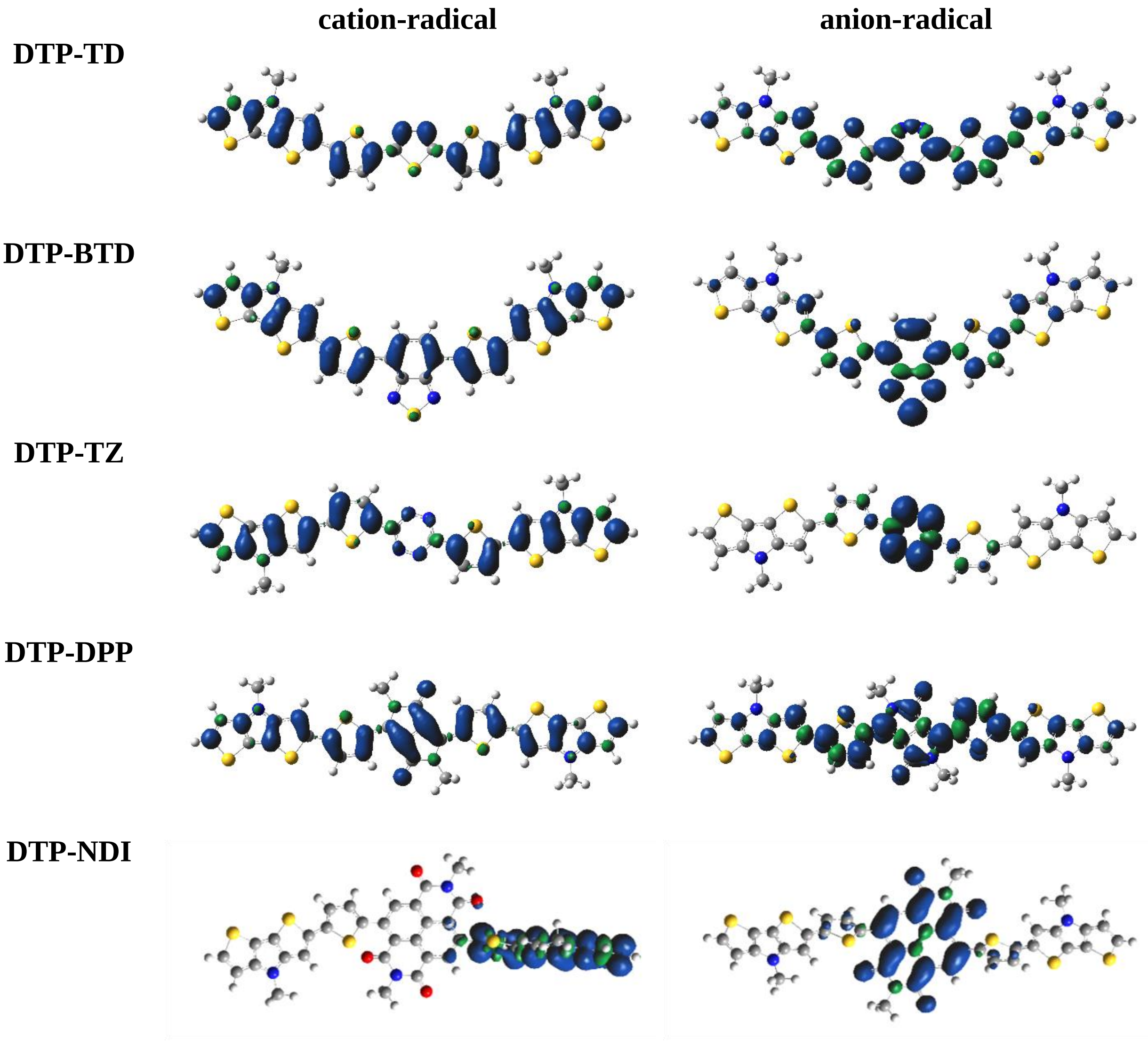

Figure S5. Isosurfaces of the spin densities of ion-radicals of the studied derivatives dissolved in **DCM,** isovalue = 0.0004 a.u. Positive spin density values are displayed in blue and negative ones in green. Method: PCM-B3LYP-D3/6-311+G*.

Table S2. Dihedral angles (in degrees) between donor–π and π–acceptor parts of the neutral molecule and both ion-radicals optimized using the (PCM-)B3LYP-D3/6-311+G* method in vacuum and in DCM solution.

Solvent /	Compound	Charge	donor–π	π–acceptor	acceptor–π	π–donor	Symmetry
Vacuum							
	**DTP-TD**	neutral	18.6	1.6	1.6	18.6	C_2
		cation	0.3	0.0	0.0	0.3	C_2
		anion	1.2	0.2	0.2	1.2	C_2
	**DTP-BTD**	neutral	18.3	9.1	9.1	18.3	C_2
		cation	0.3	0.0	0.0	0.3	C_2
		anion	6.6	0.6	0.6	6.6	C_2
	**DTP-TZ**	neutral	16.5	0.8	0.8	16.5	C_i
		cation	0.3	0.0	0.0	0.3	C_i
		anion	1.8	0.1	0.1	1.8	C_i
	**DTP-DPP**	neutral	15.9	0.6	0.6	15.9	C_i
		cation	0.4	0.1	0.1	0.4	C_i
		anion	3.6	0.3	0.3	3.6	C_i
	**DTP-NDI**	neutral	24.4	48.9	48.9	24.4	C_2
		cation	8.9	46.1	46.1	8.9	C_2
		anion	25.3	57.0	57.0	25.3	C_2
DCM							
	**DTP-TD**	neutral	10.4	2.4	2.4	10.4	C_2
		cation	1.5	1.6	1.6	1.5	C_2
		anion	0.8	0.2	0.2	0.8	C_2
	**DTP-BTD**	neutral	16.1	13.2	13.2	16.1	C_2
		cation	0.7	1.2	1.2	0.7	C_2
		anion	11.0	0.4	0.7	11.3	–
	**DTP-TZ**	neutral	11.9	0.6	0.6	11.9	C_i
		cation	1.1	2.1	2.0	0.2	–
		anion	19.1	1.2	1.2	19.1	C_i
	**DTP-DPP**	neutral	12.2	0.6	0.6	12.2	C_i
		cation	0.9	0.1	0.1	0.8	–

	anion	5.6	0.5	0.5	5.6	C_i
**DTP-NDI**	neutral	21.0	48.6	48.6	21.0	C_2
	cation	18.1	44.2	63.6	0.9	–
	anion	22.2	57.4	57.4	22.2	C_2

Table S3. Dihedral angles (in degrees) between donor–π and π–acceptor parts of the neutral molecule and both ion-radicals optimized using the (PCM-)MN15/6-311+G* method in vacuum and in DCM solution.

Solvent / Compound	Charge	donor–π	π–acceptor	acceptor–π	π–donor	Symmetry
Vacuum						
**DTP-TD**	neutral	16.3	1.1	1.1	16.3	C_2
	cation	0.2	0.0	0.0	0.2	C_2
	anion	0.6	0.1	0.1	0.6	C_2
**DTP-BTD**	neutral	16.4	10.4	10.4	16.4	C_2
	cation	0.2	0.1	0.1	0.2	C_2
	anion	5.1	0.2	0.2	5.1	C_2
**DTP-TZ**	neutral	15.6	0.6	0.6	15.6	C_i
	cation	0.2	0.1	0.1	0.2	C_i
	anion	0.9	0.0	0.0	0.9	C_i
**DTP-DPP**	neutral	14.5	2.2	2.3	16.6	–
	cation	0.3	0.1	0.0	0.2	–
	anion	1.4	0.1	0.1	1.3	–
**DTP-NDI**	neutral	21.8	48.6	48.6	21.8	C_2
	cation	5.3	42.0	42.0	5.3	C_2
	anion	22.6	53.9	53.9	22.6	C_2
DCM						
**DTP-TD**	neutral	3.6	0.4	0.4	3.6	C_2
	cation	1.4	0.4	0.4	1.4	C_2
	anion	0.3	0.1	0.1	0.3	C_2
**DTP-BTD**	neutral	14.2	16.1	16.1	14.2	C_2
	cation	0.4	4.0	3.1	0.2	–
	anion	8.6	0.8	0.6	9.3	–

**DTP-TZ**	neutral	11.5	0.3	0.3	11.5	C_i
	cation	0.7	0.2	0.2	0.7	C_i
	anion	17.4	0.7	0.7	17.4	C_i
**DTP-DPP**	neutral	7.5	2.3	2.1	7.2	–
	cation	0.8	0.1	0.1	0.8	–
	anion	1.7	1.0	1.0	1.6	–
**DTP-NDI**	neutral	19.6	49.2	49.2	19.6	C_2
	cation	17.9	47.2	65.3	0.6	–
	anion	19.1	54.7	54.7	19.1	C_2

Table S4. Dihedral angles (in degrees) between donor–π and π–acceptor parts of the neutral molecules in the relaxed S_1 state in vacuum and in DCM solution optimized using the TDA-(PCM-)MN15/6-311+G* method.

Solvent /	Compound	donor–π	π–acceptor	acceptor–π	π–donor	Symmetry
Vacuum						
	**DTP-TD**	0.2	0.0	0.0	0.2	C_2
	**DTP-BTD**	0.7	0.2	0.2	0.7	C_2
	**DTP-TZ**	16.3	0.7	0.7	16.3	C_i
	**DTP-DPP**	0.3	0.0	0.0	0.3	–
	**DTP-NDI**	25.8	56.1	89.2	1.7	–
DCM						
	**DTP-TD**	0.8	0.3	0.3	0.8	C_2
	**DTP-BTD**	1.7	0.5	0.5	1.7	C_2
	**DTP-TZ**	12.0	0.5	0.5	12.0	C_i
	**DTP-DPP**	0.4	0.1	0.1	0.4	–
	**DTP-NDI**	20.3	58.6	89.5	1.3	–

5. Electropolymerization

a)

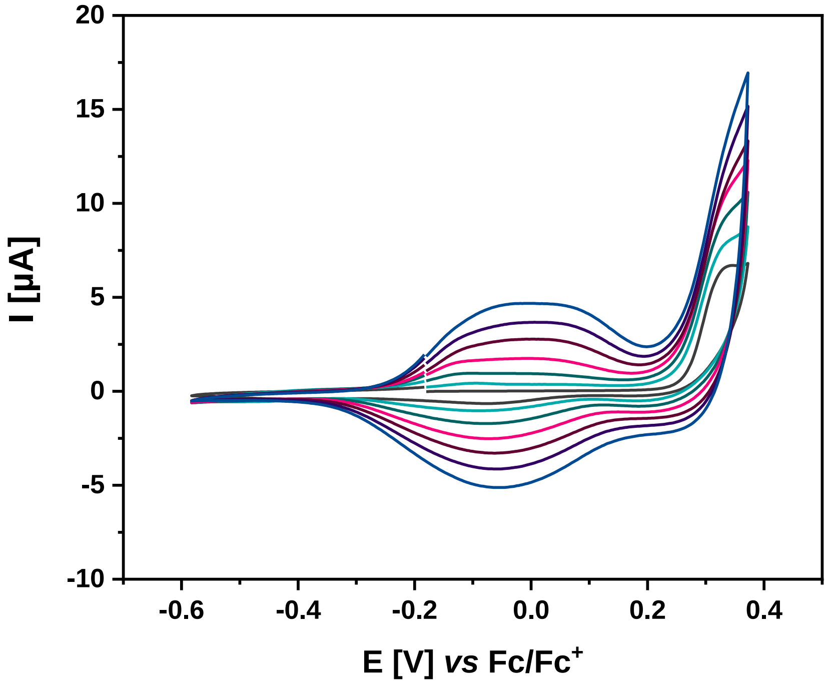

b)

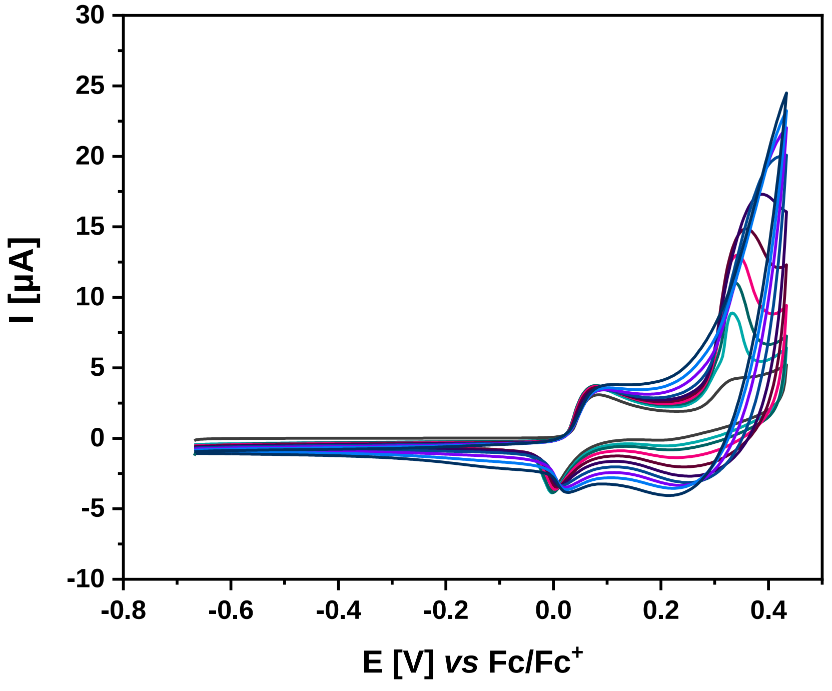

c)

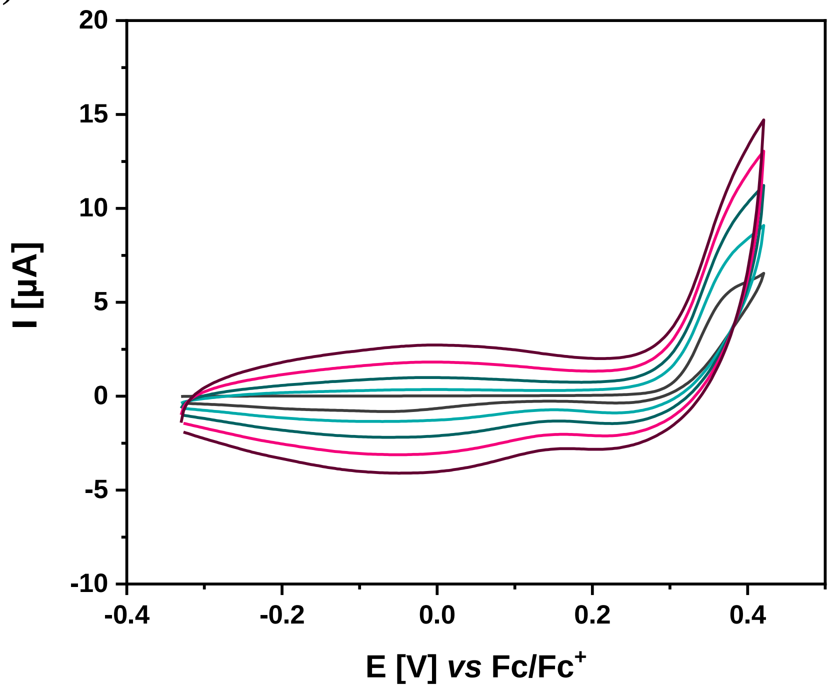

d)

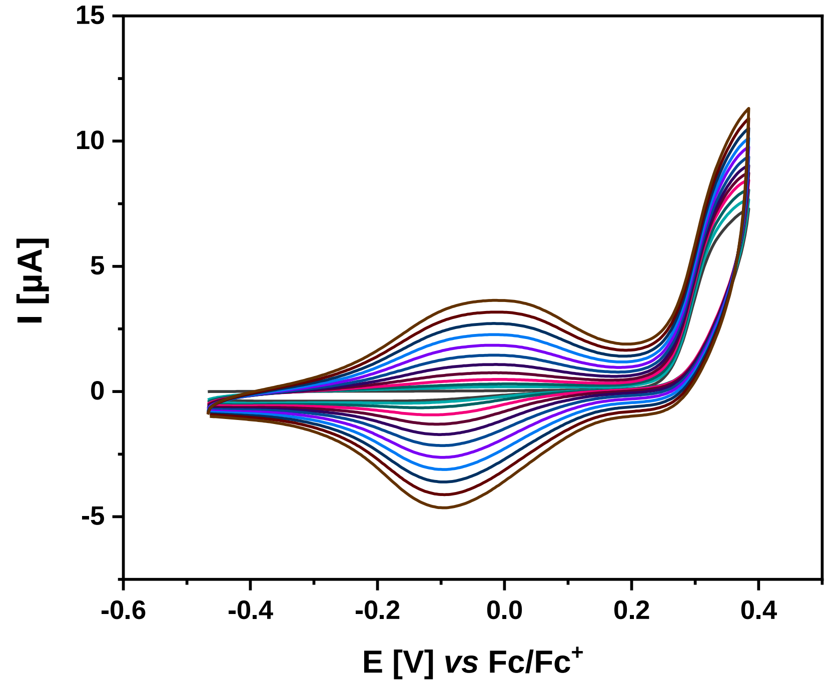

Figure S6. Consecutive CV scans registered during electropolymerization of **DTP-TD** (a), **DTP-BDT** (b), **DTP-TZ** (c) and **DTP-NDI** (d). Platinum disk electrode, electrolyte: 0.1 M Bu_4NP_6/DCM, scan rate = 50 mV/s.

6. Electrochromism

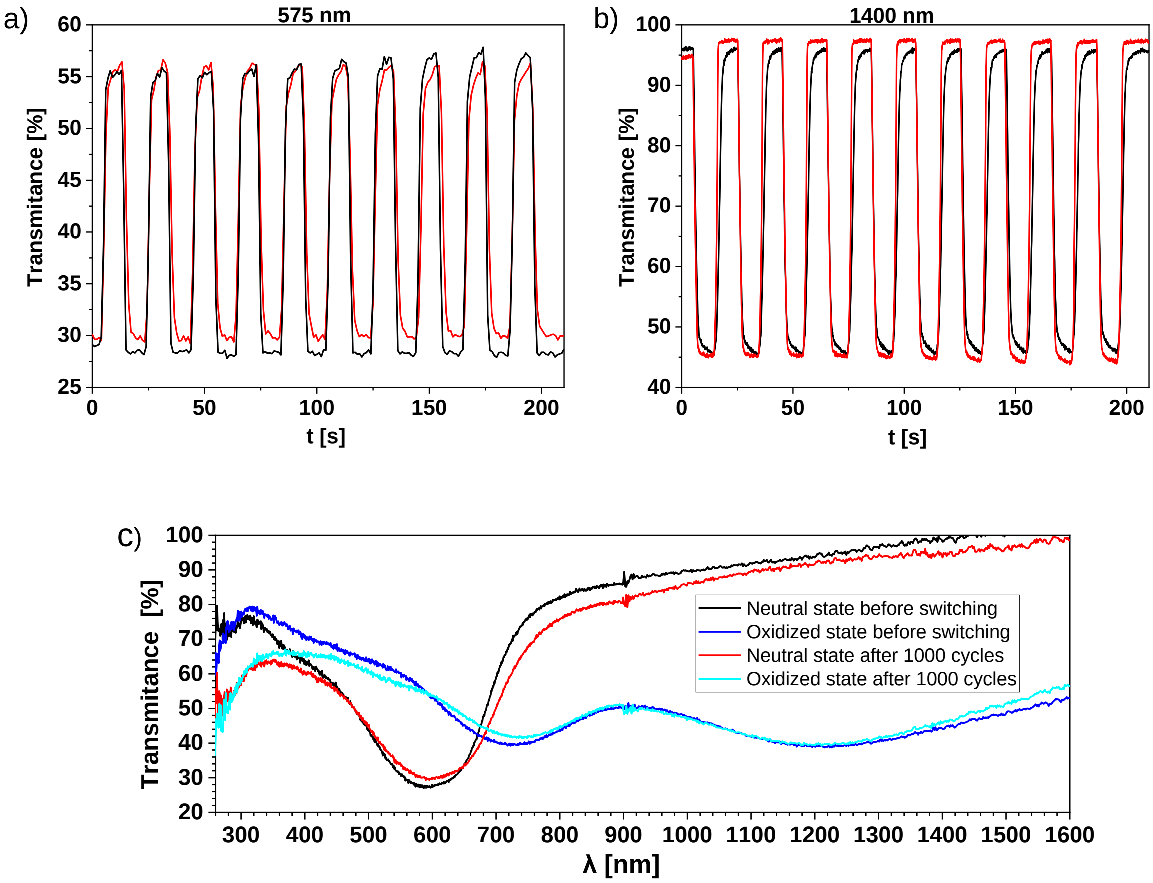

Figure S7. Transmittance changes induced by electrochromic switching of **poly(DTP-DT)**, recorded in the beginning of the switching test (black curves) and after 1000 switching cycles (red curves), recorded for (a) 575 nm and (b) 1400 nm wavelengths; (c) transmittance spectra registered before switching and after 1000 switching cycles.

7. References

1. Kurach, E.; Kotwica, K.; Zapala, J.; Knor, M.; Nowakowski, R.; Djurado, D.; Toman, P.; Pfleger, J.; Zagorska, M.; Pron, A. Semiconducting Alkyl Derivatives of 2,5-Bis(2,2′-Bithiophene-5-Yl)-1,3,4-Thiadiazole—Effect of the Substituent Position on the Spectroscopic, Electrochemical, andStructural Properties. *J. Phys. Chem. C* **2013**, *117*, 15316–15326.
2. Fell, V. H. K.; Findlay, N. J.; Breig, B.; Forbes, C.; Inigo, A. R.; Cameron, J.; Kanibolotsky, A. L.; Skabara, P. J. Effect of End Group Functionalisation of Small Molecules Featuring the Fluorene–Thiophene–Benzothiadiazole Motif as Emitters in Solution-Processed

Red and Orange Organic Light-Emitting Diodes. *J. Mater. Chem. C* **2019**, *7*, 3934–3944.

3. Kurach, E.; Djurado, D.; Rimarčik, J.; Kornet, A.; Wlostowski, M.; Lukeš, V.; Pécaut, J.; Zagorska, M.; Pron, A. Effect of Substituents on Redox, Spectroscopic and Structural Properties of Conjugated Diaryltetrazines—a Combined Experimental and Theoretical Study. *Phys. Chem. Chem. Phys.* **2011**, *13*, 2690–2700.
4. Gora, M.; Krzywiec, W.; Mieczkowski, J.; Rodrigues Maia, E. C.; Louarn, G.; Zagorska, M.; Pron, A. Alternating Copolymers of Diketopyrrolopyrrole orBenzothiadiazole and Alkoxy-Substituted Oligothiophenes: Spectroscopic, Electrochemical and Spectroelectrochemical Investigations. *Electrochim. Acta* **2014**, *144*, 211–220.
